# The Endoplasmic Reticulum ATP13A1 is Essential for MAVS‐Mediated Antiviral Innate Immunity

**DOI:** 10.1002/advs.202203831

**Published:** 2022-10-10

**Authors:** Rui Zhang, Xianteng Hou, Changwan Wang, Jiaxin Li, Junyan Zhu, Yingbo Jiang, Fajian Hou

**Affiliations:** ^1^ State Key Laboratory of Molecular Biology Shanghai Institute of Biochemistry and Cell Biology Center for Excellence in Molecular Cell Science Chinese Academy of Sciences University of Chinese Academy of Sciences Shanghai 200031 China; ^2^ Key Laboratory of Systems Health Science of Zhejiang Province School of Life Science Hangzhou Institute for Advanced Study University of Chinese Academy of Sciences Hangzhou 310024 China

**Keywords:** ATP13A1, innate immunity, MAVS, protein degradation, signaling transduction

## Abstract

RIG‐I‐MAVS signaling pathway is essential for efficient innate immune response against virus infection. Though many components have been identified in RIG‐I pathway and it can be partially reconstituted in vitro, detailed mechanisms involved in cells are still unclear. Here, a genome‐wide CRISPR‐Cas9 screen is performed using an engineered cell line *IFNB‐P2A‐GSDMD‐N*, and ATP13A1, a putative dislocase located on the endoplasmic reticulum, is identified as an important regulator of RIG‐I pathway. *ATP13A1* deficiency abolishes RIG‐I‐mediated antiviral innate immune response due to compromised MAVS stability and crippled signaling potency of residual MAVS. Moreover, it is discovered that MAVS is subject to protease‐mediated degradation in the absence of ATP13A1. As homozygous *Atp13a1* knockout mice result in developmental retardation and embryonic lethality, *Atp13a1* conditional knockout mice are generated. Myeloid‐specific *Atp13a1‐*deficient mice are viable and susceptible to RNA virus infection. Collectively, the findings reveal that ATP13A1 is indispensable for the stability and activation of MAVS and a proper antiviral innate immune response.

## Graphical abstract

ER‐localized ATP13A1 is identified as an essential regulator of RIG‐I‐MAVS signaling via an unbiased genome‐wide screen. Mechanistically, ATP13A1 interacts with MAVS and is essential for its proper localization and antiviral activity. Deficiency of ATP13A1 results in protease‐mediated degradation of MAVS.

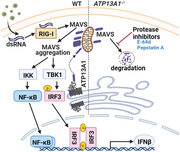

## 1 Introduction

Innate immunity is the first line of defense against microbial pathogens. Innate immune response mediated by distinct signaling pathways is initiated upon recognition of pathogen‐associated molecular patterns (PAMPs) or damage‐associated molecular patterns (DAMPs) by pattern recognition receptors (PRRs).^[^ ^1^ ^]^ As one of the key cytoplasmic sensors, RIG‐I is activated and triggers MAVS (also known as VISA, IPS‐1, and Cardif) to aggregate once it detects pathogenic or aberrant RNA.^[^ ^2^ ^]^ Aggregated MAVS promotes a signaling cascade subsequently to turn on the expression of type I interferons (IFN‐I) and inflammatory cytokines. Defective RIG‐I‐MAVS signaling prevents a proper innate immune response and pathogen clearance.^[^ ^3^ ^]^ However, excessive activation of IFN‐I signaling leads to autoimmune diseases. Thus, the RIG‐I‐MAVS signaling pathway is tightly regulated to maintain immune homeostasis of the host.

Post‐translational modifications play a key role in the regulation of RIG‐I‐MAVS signaling. E3 ligase Riplet, along with TRIM25, is responsible for K63‐linked ubiquitination of RIG‐I, and potentiates its activation.^[^ ^4^ ^]^ Deubiquitinases including USP3, USP21, and CYLD attenuate the signaling from RIG‐I to MAVS.^[^ ^5^ ^]^ Dephosphorylation of serine site by PP1*α* and PP1*γ* also contributes to the activation of RIG‐I.^[^ ^6^ ^]^ TRIM31 promotes MAVS aggregation through K63‐linked ubiquitination, while FAF1 and OTUD3 regulate the pathway oppositely.^[^ ^7^ ^]^ TRAF3IP3 facilitates the recruitment of TRAF3 to MAVS and serves as a positive regulator upon virus infection.^[^ ^8^ ^]^ In addition, E3 ligases, such as RNF5, RNF125, MARCH5, and pVHL mediate K48‐linked ubiquitination and degradation of MAVS to inhibit interferon production.^[^ ^9^ ^]^ Lactate, a product of glucose metabolism, inhibits the antiviral response by affecting MAVS localization through binding with its transmembrane domain (TM) and preventing the interaction of RIG‐I with MAVS.^[^ ^10^ ^]^ In contrast, interaction of cytosolic phospholipase A2 with MAVS potentiates activation of NF‐*κ*B and pro‐inflammatory transcriptions in astrocytes.^[^ ^11^ ^]^ Moreover, internal regions of MAVS restrict its activity by an autoinhibitory mechanism and N‐terminally truncated isoforms of MAVS prevent its spontaneous aggregation by interacting with TM domain of full‐length MAVS in unstimulated cells.^[^ ^12^ ^]^

P‐type ATPases are widely expressed membrane proteins that hydrolyze ATP and transport ions or other substrates such as phospholipids, across biological membranes, accompanying phosphorylation and dephosphorylation of a conserved aspartate during the transport cycle.^[^ ^13^ ^]^ The family comprises five subfamilies (P1–P5) based on phylogenetic analysis. P1–P3 ATPases are cation transporters and P4 ATPases are involved in lipid flipping.^[^ ^14^ ^]^ In contrast to P1–P4 ATPases with definite substrates and molecular functions, P5 ATPases are poorly characterized. P5 ATPases are found in almost all eukaryotes and can be divided into P5A and P5B subgroups based on DNA sequence alignment. P5A subgroup contains only one member in human and mice, named ATP13A1 or Atp13a1 respectively, while ATP13A2–A5 (or Atp13a2–a5) constitute P5B subgroup. The most well‐characterized P5A‐ATPase is Cod1/Spf1 in the yeast, and previous studies have shown that it participates in various biological processes such as protein folding and processing, sterol synthesis and transport, manganese or calcium transport, tail‐anchored protein insertion, and protein glycosylation.^[^ ^15^ ^]^ The expression of P5 ATPases is likely developmentally regulated and their distribution varies in different organs of mice.^[^ ^16^ ^]^ Mutations of ATP13A2 and ATP13A4 are probably related to some diseases in nervous system, such as Parkinson's disease and autism spectrum disorders.^[^ ^17^ ^]^ While the function of P5A‐ATPase as transmembrane helix dislocase to correct misinserted transmembrane helices from the endoplasmic reticulum (ER) have been shown recently in vitro,^[^ ^18^ ^]^ its physiological substrates and biological functions remain to be further defined.

In this study, we provide the first evidence on the important physiological function of ATP13A1 in antiviral innate immune response. Loss of *ATP13A1* attenuated the expression of antiviral genes upon virus infection. Mechanistically, antiviral protein MAVS was degraded by proteases and its activity was severely compromised as a result of *ATP13A1* deficiency, which led to compromised antiviral immune response. Homozygous *Atp13a1* knockout mice showed retarded development and embryonic lethality. Mice with myeloid‐specific *Atp13a1* deficiency showed severely impaired interferon production upon various stimulations and were vulnerable to RNA virus infection. Collectively, the study uncovers that ATP13A1 serves as an important positive regulator of antiviral innate immunity.

## 2 Results

### 2.1 Identification of ATP13A1 as a Component in Antiviral Signaling

**Figure 1:**
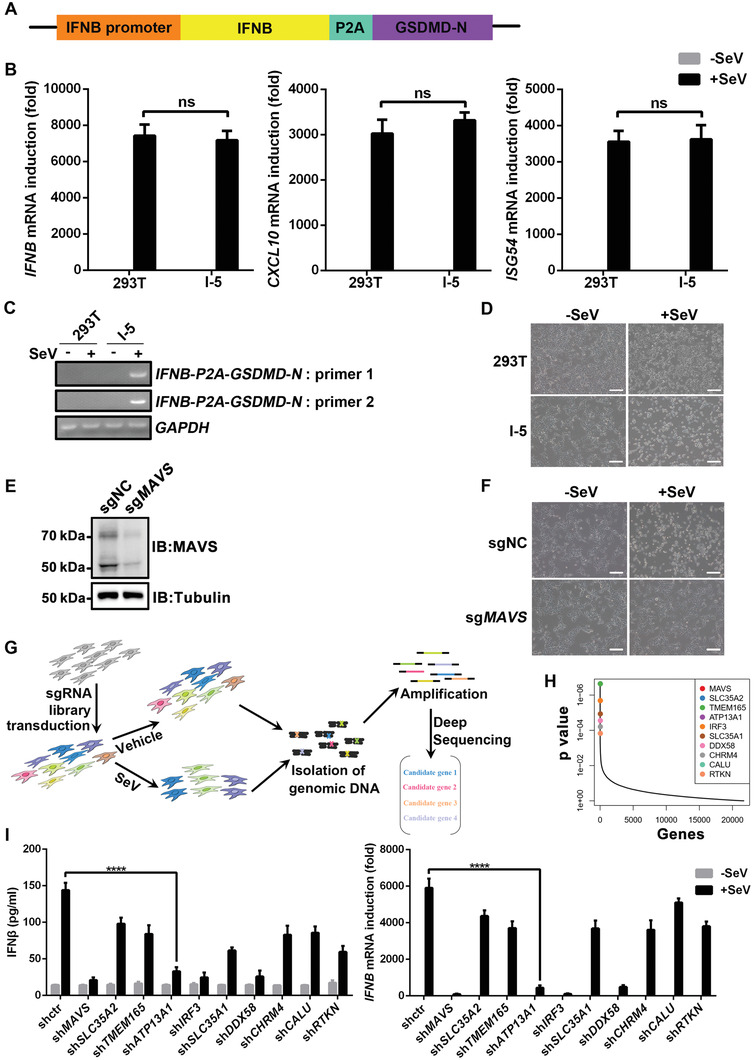
Identification of ATP13A1 involved in antiviral signaling. A) A diagram of the recombination construct used for generation of I‐5 cells. B) *IFNB*, *CXCL10* and *ISG54* induction in HEK293T cells and I‐5 cells upon SeV infection for 12 h. C) Transcription of *GSDMD*‐*N* in HEK293T cells and I‐5 cells upon SeV infection. D) HEK293T cells and I‐5 cells were infected with SeV for 12 h before microscopic imaging. E) Knockdown efficiency of MAVS by sgRNA in I‐5 cells stabling expressing Cas9 (I‐5(Cas9)). F) Microscopic imaging of I‐5 (Cas9) cells with or without SeV infection for 12 h. G) Outline of the genome‐scale CRISPR‐Cas9 screening. H) P‐value of the hits identified from the screening. I) shRNA as indicated were transfected into HEK293T cells, which were infected with or without SeV for 12 h before ELISA and qPCR analysis. Data are representative of three independent experiments (shown as mean and SD in B,I). *p* value was determined by two‐tailed unpaired Student's *t*‐test. ****p < 0.0001. ns indicates not statistically significant.

To identify potential regulators of RIG‐I‐MAVS signaling pathway, we generated a cell line *IFNB‐P2A‐GSDMD‐N* (namely I‐5), in which a N‐terminal fragment of Gasdermin D (GSDMD‐N) was knocked‐in and fused with the endogenous *IFNB* gene in HEK293T cells (**Figure** **1**A). Accordingly, when *IFNB* is induced in I‐5 cells upon stimulation, GSDMD is concomitantly expressed as a fusion protein with IFN*β*, which is subject to protease cleavage targeting a P2A cleaving site between GSDMD and IFN*β*. As shown in Figure 1B, upon Sendai virus (SeV) stimulation in I‐5 cells, we detected transcription of antiviral genes, such as *IFNB*, *CXCL10*, and *ISG54*, which is comparable to those in wild‐type HEK293T cells, suggesting that I‐5 cells respond normally to virus infection by activation of RIG‐I pathway. Meanwhile, I‐5 cells but not wild‐type HEK293T cells express GSDMD‐N upon SeV infection (Figure 1C). GSDMD‐N is known to be able to trigger pyroptosis, a lytic‐regulated cell death. Consistently, I‐5 cells, but not the wild‐type HEK293T cells, undergo cell death upon SeV stimulation (Figure 1D).

To further validate that the “death‐upon‐stimulation” phenotype of I‐5 cells is due to RIG‐I pathway, MAVS was knocked down by sgRNA, after which I‐5 cells did not undergo pyroptosis and were viable upon SeV infection (Figure 1E,F). This result indicates that the “death‐upon‐stimulation” phenotype is dependent on MAVS and is a valid readout of RIG‐I‐MAVS signaling. We then utilized the I‐5 cells for a CRISPR‐Cas9 screening with the rationale that cells will die upon SeV infection except those with deficiency in key components of the RIG‐I pathway. The screening was performed as described in the flowchart (Figure 1G). Surviving cells were collected and sgRNA‐targeted genes were sequenced (Figure 1H). Among the top hits are known players in RIG‐I pathway, such as *MAVS*, *IRF3*, and *DDX58*. We selected a few candidates, which have not been reported to be involved in RIG‐I pathway, and further examined them in HEK293T cells by loss‐of‐function assay. Notably, knockdown of ATP13A1, a previously identified ER‐localized dislocase, substantially dampened *IFNB* induction upon SeV infection (Figure 1I). These results suggested that ATP13A1 might be an essential player in RIG‐I pathway and we decided to dissect the mechanism involved in the ER protein ATP13A1‐mediated antiviral signaling.

### 2.2 ATP13A1 Positively Regulates RIG‐I‐MAVS Signaling Pathway

**Figure 2:**
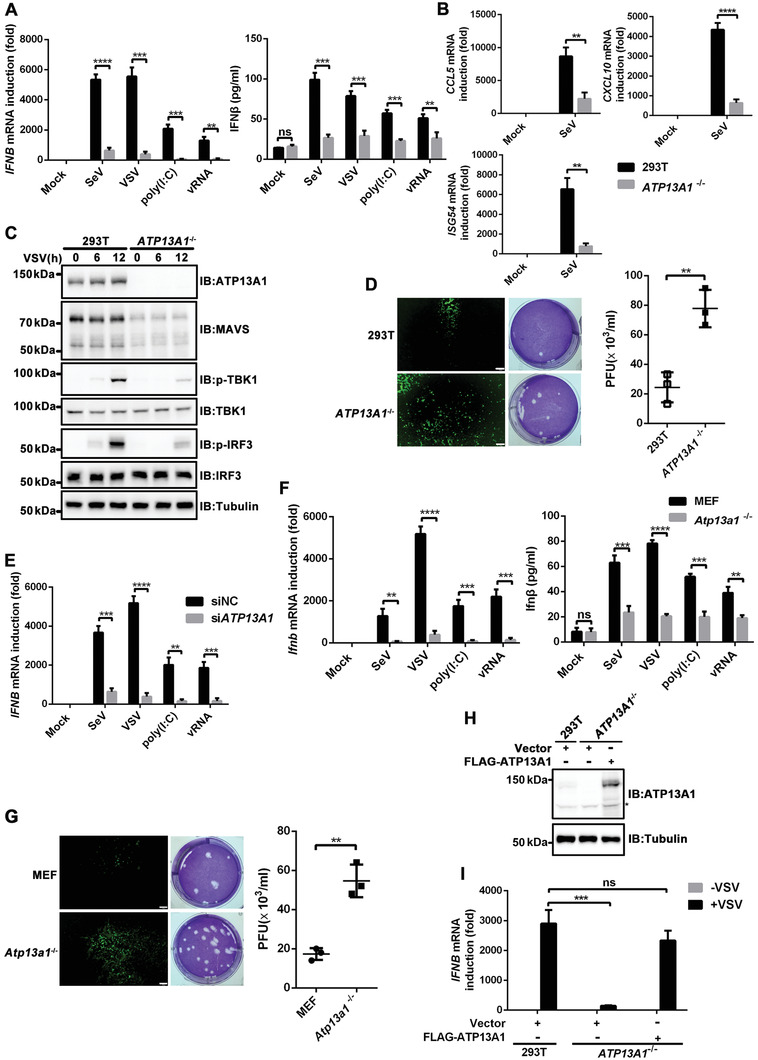
ATP13A1 positively regulates RIG‐I‐MAVS signaling pathway. A) qPCR and ELISA analysis for the induction and expression of *IFNB* in wild‐type or *ATP13A1* ^−/−^ HEK293T cells with various stimuli as indicated. vRNA refers to genomic RNA isolated from VSV. B) Induction of *CCL5*, *CXCL10* and *ISG54* in wild‐type and *ATP13A1* ^−/−^ HEK293T cells with or without SeV infection. C) Immunoblotting analysis of cell lysates from wild‐type or *ATP13A1* ^−/−^ HEK293T cells with VSV infection for the indicated time. D) Wild‐type or *ATP13A1* ^−/−^ HEK293T cells were infected with VSV‐GFP (MOI = 0.1) for 12 h before microscopic imaging (left) and plaque assay (right). E) qPCR analysis for the transcription of *IFNB* in wild‐type and *ATP13A1*‐knockdown HEK293T cells with various stimuli as indicated. F) qPCR and ELISA analysis for the transcription and expression of *Ifnb* in wild‐type and *Atp13a1* ^−/−^ MEF cells with various stimuli as indicated. G) Wild‐type or *Atp13a1* ^−/−^ MEF cells were infected with VSV‐GFP (MOI = 0.1) for 12 h before microscopic imaging (left) and plaque assay (right). H) Immunoblotting analysis for FLAG‐ATP13A1 expression in *ATP13A1* ^−/‐^ HEK293T cells. Asterisk indicated nonspecific bands. I) qPCR analysis for the induction of *IFNB* in HEK293T cells following transient expression of the indicated plasmids. Data are representative of three independent experiments (shown as mean and SD in (A,B,D–G,I)). *p* value was determined by two‐tailed unpaired Student's *t*‐test, **p < 0.01, ***p < 0.001, ****p < 0.0001. ns indicates not statistically significant.

*ATP13A1* was knocked‐out in HEK293T cells. Strikingly, *IFNB* induction at both mRNA and protein levels was severely attenuated in *ATP13A1* ^−/−^ cells upon stimulation with various stimuli (**Figure** **2**A; Figure S1A, Supporting Information). In addition, the transcription of antiviral genes, including *CCL5*, *CXCL10*, and *ISG54*, was also potently inhibited in the absence of ATP13A1 (Figure 2B). Consistently, TBK1 phosphorylation and IRF3 phosphorylation were greatly diminished in *ATP13A1* ^−/‐^ cells (Figure 2C). Moreover, *ATP13A1* deficiency potentiated the proliferation of VSV‐GFP in HEK293T cells, suggesting that ATP13A1 is essential for an effective antiviral defense (Figure 2D). The dampened response to SeV, VSV, poly(I:C), and VSV RNA (vRNA) stimulation was also evident in HEK293T cells when *ATP13A1* was knocked down (Figure 2E; Figure S1A, Supporting Information). Furthermore, the essential role of *Atp13a1* in *Ifnb* induction was also validated in MEF cells (Figure 2F; Figure S1A, Supporting Information). Consistently, reduced viral clearance was observed in *Atp13a1* ^−/−^ MEF cells (Figure 2G). Notably, the defect of *IFNB* induction was restored in *ATP13A1* ^−/‐^ HEK293T cells by exogenous expression of wild‐type ATP13A1 but not the catalytically inactive mutant or other truncated mutants (Figure 2H,I; Figure S1B–F, Supporting Information).^[^ ^18b^ ^]^ These results suggested that ATP13A1 is indeed essential in antiviral signaling.

ATP13A1 is a multiple transmembrane protein located on the ER and a member of P‐type ATPases. Previous studies suggested that Spf1, the yeast homology of ATP13A1, is essential to maintain the homeostasis of the ER. Therefore, ATP13A1 deficiency might result in ER stress and unfolded protein response (UPR), which could interact with RIG‐I‐MAVS signaling pathway. We then analyzed the induction of downstream genes of three major UPR effectors, i.e., PERK, IRE1*α*, and ATF6 in *ATP13A1* ^−/−^ HEK293T cells. No difference in expression of UPR‐dependent genes was found between *ATP13A1* ^−/−^ cells and wild‐type cells treated with or without thapsigargin, a known small molecule that could induce UPR (Figure S2A, Supporting Information). Similar results were obtained when *ATP13A1* was knocked down (Figure S2B, Supporting Information). Additionally, the IRE1*α* signaling pathway is known to cleave off a 26‐mer nucleotide in the stem ring of *XBP1* upon ER stress, generating an active form. We could not detect the spliced form of *XBP1* in *ATP13A1* ^−/‐^ HEK293T cells and *Atp13a1* ^−/‐^ MEF cells unless treated with thapsigargin (Figure S2C,D, Supporting Information). These results collectively suggested that the antiviral function of ATP13A1 in RIG‐I pathway is independent on UPR pathway.

### 2.3 ATP13A1 is Required for Maintenance of Proper MAVS Protein Level

**Figure 3:**
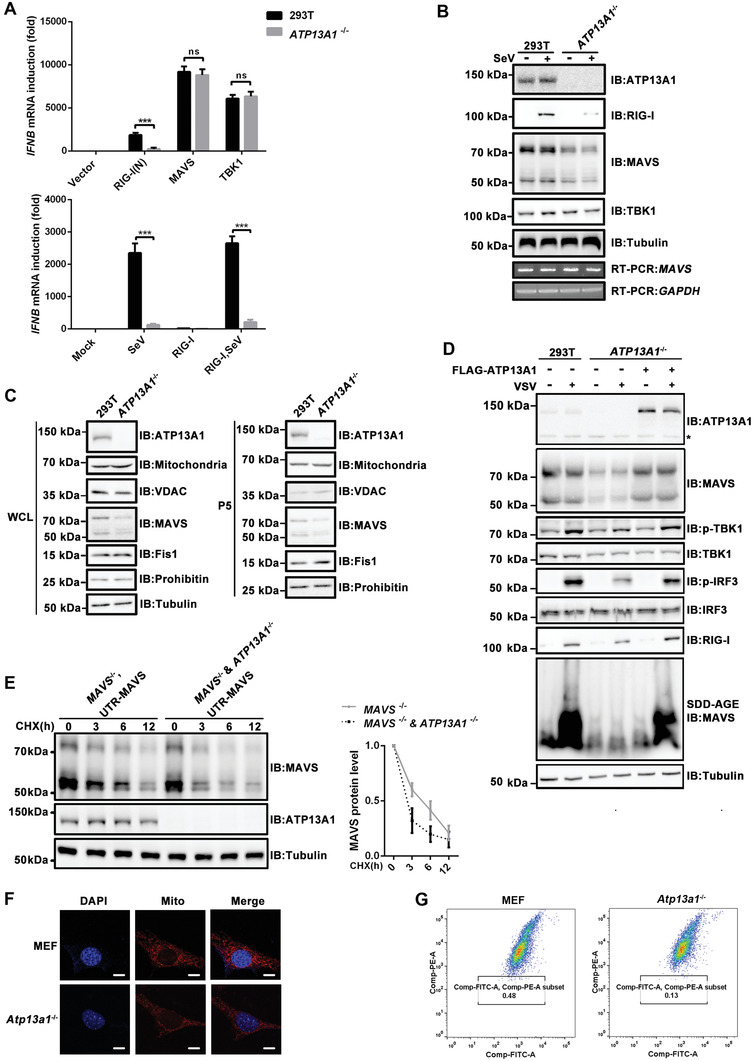
ATP13A1 is required for maintenance of proper MAVS protein level. A) Various plasmids were transfected into wild‐type or *ATP13A1* ^−/−^ HEK293T cells. Twenty‐four hours after transfection, the cells were infected with SeV for 12 h before qPCR analysis for the induction of *IFNB*. B) Wild‐type and *ATP13A1* ^−/−^ HEK293T cells were infected with SeV for 12 h. Whole cell lysates were then harvested for immunoblotting analysis. C) Whole cell lysates and P5 fractions from wild‐type or *ATP13A1* ^−/−^ HEK293T cells were analyzed by immunoblotting. D) *ATP13A1* ^−/−^ HEK293T cells were transfected with FLAG‐ATP13A1‐expressing vectors for 24 h. The cells were then infected with VSV for 12 h before immunoblotting analysis. Asterisk indicated nonspecific bands. E) *MAVS* ^−/−^ and *MAVS^−/−^* & *ATP13A1^−/−^* double knockout HEK293T cells were transfected with UTR‐MAVS for 24 h, and then treated with CHX for the indicated time before immunoblotting analysis (left). Protein level of MAVS normalized to tubulin was quantified (right). F) Wild‐type and *Atp13a1* ^−/−^ MEF cells were stained for immunofluorescent microscopic imaging. Nuclei were stained with DAPI. Mitochondria were stained with MitoTracker Red. Scale bar represents 10 micrometers. G) Flow cytometry analyses of mitochondrial membrane potential in wild‐type and *Atp13a1* ^−/−^ MEF cells with JC‐1 MitoMP Detection Kit. Data are representative of three independent experiments (shown as mean and SD in (A), mean and SEM in (E)). *p* value was determined by two‐tailed unpaired Student's *t*‐test, ***p< 0.001. ns indicates not statistically significant.

We went on to investigate which known component of the RIG‐I‐MAVS pathway might be disrupted in the absence of ATP13A1. First, we overexpressed RIG‐I (N), RIG‐I, MAVS, and TBK1 in the wild‐type and *ATP13A1* ^−/−^ HEK293T cells respectively, which are known to be able to induce *IFNB* production assuming an intact downstream signaling. Compared to the wide type cells, *ATP13A1*‐deficienct cells showed reduced *IFNB* production, when RIG‐I (N) or RIG‐I but not MAVS or TBK1, was overexpressed. These data suggested that ATP13A1 plays a role downstream of RIG‐I and upstream of MAVS (**Figure** **3**A; Figure S3A, Supporting Information). We then analyzed the expression and status of known effectors in RIG‐I pathway, and to our surprise, MAVS protein was substantially reduced in *ATP13A1* ^−/−^ HEK293T cells regardless of stimulation (Figure 3B). Downregulation of MAVS is not at transcriptional level as MAVS mRNA expression was not affected by ATP13A1 deficiency. We also examined other proteins from either crude cell lysate or isolated mitochondria fraction only to find that MAVS was the sole protein with reduced expression in *ATP13A1‐*deficienct cells (Figure 3C; Figure S3B,C, Supporting Information). In addition, the reduction of MAVS protein was found in both whole cell lysate and mitochondrial fraction, but not in mitochondria‐associated membranes (MAMs) fraction, which were indicative of different targeting and membrane insertion pathways (Figure S3D, Supporting Information). Strikingly, the reduction of MAVS could be rescued when ectopic ATP13A1 was transiently expressed in *ATP13A1* ^−/−^ cells (Figure 3D). Meanwhile, the activation of MAVS, TBK1, and IRF3 was also restored upon stimulation. These data indicated that MAVS reduction was indeed due to the absence of ATP13A1 but not an off‐target effect in *ATP13A1‐*deficienct cells. We then deduced that ATP13A1 might be required for MAVS stability. In addition, we transiently expressed MAVS or MAVS (ΔTM) mutant containing 5'‐UTR at a near‐physiological level in *MAVS* ^−/−^ and *MAVS* ^−/−^ & *ATP13A1* ^−/−^ cells. We then treated cells with cycloheximide (CHX), an inhibitor to protein synthesis, to examine MAVS stability. We found that full‐length MAVS was degraded much faster in the absence of ATP13A1, suggesting that ATP13A1 is indeed required for MAVS stability (Figure 3E). Meanwhile, MAVS (ΔTM) mutant in *MAVS* ^−/−^ cells, which lacks the transmembrane domain, was as stable as that in *MAVS* ^−/−^ & *ATP13A1* ^−/‐^ cells, suggesting that the membrane localization is involved in MAVS stability (Figure S3E, Supporting Information). Moreover, mitochondrial morphology in *ATP13A1‐*deficienct cells was normal and similar to wild‐type cells (Figure 3F). Mitochondrial membrane potential in *ATP13A1‐*deficienct cells was examined and revealed no difference from wild‐type cells. These results suggested that mitochondria were intact and not damaged in the absence of ATP13A1 (Figure 3G). Taken together, these data indicated that ATP13A1 is required to maintain MAVS protein level without disrupting mitochondrial integrity.

### 2.4 MAVS is Degraded by Proteases in the Absence of ATP13A1

**Figure 4:**
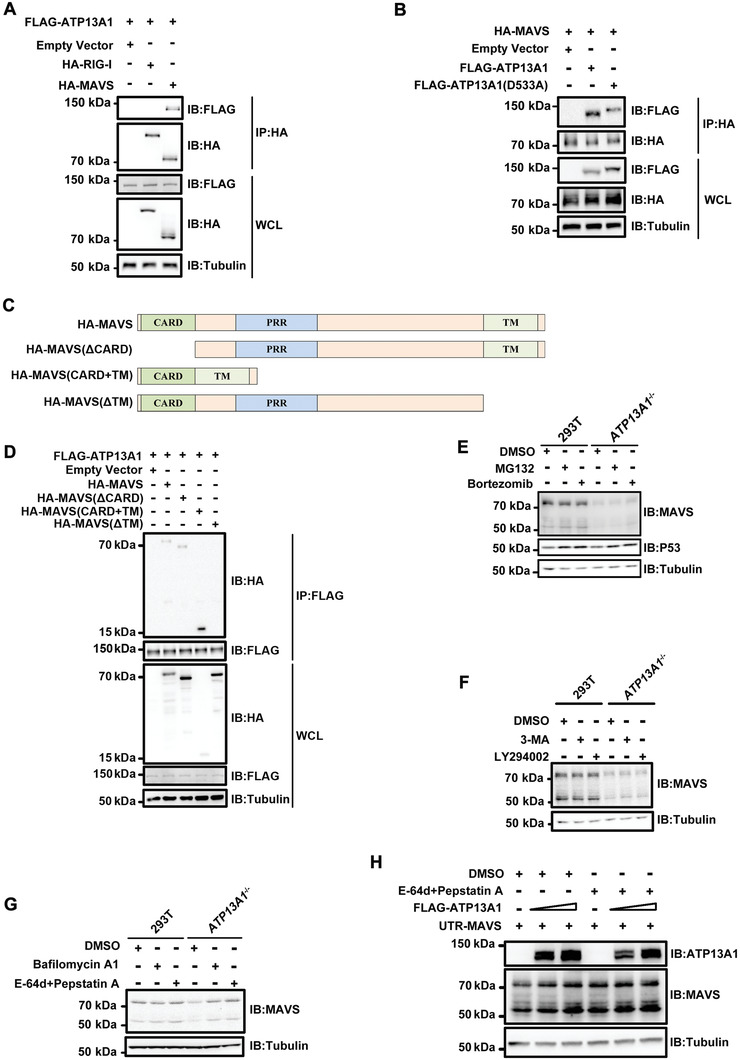
MAVS is degraded by proteases in the absence of ATP13A1. A) HA‐MAVS or HA‐RIG‐I were co‐expressed with FLAG‐ATP13A1 in HEK293T cells for 36 h before immunoprecipitation and immunoblotting. B) FLAG‐ATP13A1 or FLAG‐ATP13A1 (D533A) were co‐expressed with HA‐MAVS in HEK293T cells for 36 h before immunoprecipitation and immunoblotting. C) Diagram of MAVS truncations used in Co‐IP experiments. D) Various MAVS truncations were co‐expressed with FLAG‐ATP13A1 in HEK293T cells for 36 h before immunoprecipitation and immunoblotting. E) Wild‐type and *ATP13A1* ^−/−^ HEK293T cells were treated with MG132 (10 µm) or Bortezomib (10 µm) for 6 h before immunoblotting analysis. F) Wild‐type and *ATP13A1* ^−/−^ HEK293T cells were treated with 3‐MA (5 mm) or LY294002 (10 µm) for 6 h before immunoblotting analysis. G) Wild‐type and *ATP13A1* ^−/−^ HEK293T cells were treated with Bafilomycin A1 (200 nm), E‐64d (10 µg ml^−1^) or Pepstatin A (10 µg ml^−1^) for 12 h before immunoblotting analysis. H) *MAVS^−/−^* & *ATP13A1^−/−^* double knockout HEK293T cells were transfected with UTR‐MAVS, along with empty vector or FLAG‐ATP13A1 for 24 h, before treated with indicated inhibitors for 12 h followed by immunoblotting analysis.

We next investigated whether ER‐localized ATP13A1 interacts with mitochondria‐localized MAVS (Figure S4A, Supporting Information). MAVS but not RIG‐I can be co‐immunoprecipitated with ATP13A1, suggesting an interaction between MAVS and ATP13A1 (**Figure** **4**A). Co‐immunoprecipitation experiment also revealed that MAVS binds to ATP13A1 with a point mutation (D533A) that presumably disrupts ATP13A1 activity, suggesting that ATP13A1 interacts with MAVS independent of its catalytic activity (Figure 4B). To map the interacting domains between MAVS and ATP13A1, we generated a series of truncated MAVS and found that MAVS TM domain is required for its interaction with ATP13A1 (Figure 4C,D). Interestingly, both the cytoplasmic fragment and TM domain of ATP13A1 could bind to MAVS (Figure S4B‐D, Supporting Information).

We further explored the degradation mechanism of MAVS in *ATP13A1* ^−/‐^ cells. While treatment of *ATP13A1*‐deficienct cells with proteasome inhibitors (MG132 or Bortezomib) led to the accumulation of P53, which is known to be degraded by the proteasomal pathway, MAVS level was not restored (Figure 4E). Likewise, treatment with 3‐MA and LY294002, two inhibitors that can block autophagosome formation, could not restore MAVS level, despite that they stabilized P62 following EBSS‐induced autophagy (Figure 4F; Figure S4E, Supporting Information). Similarly, blocking autophagy‐mediated degradation by knocking‐down ATG5, an essential component involved in autophagy, failed to restore MAVS level (Figure S4F, Supporting Information). In contrast, treatment of *ATP13A1* ^−/‐^ cells with inhibitors to lysosome acidification (Bafilomycin A1 or Hydroxychloroquine) or protease inhibitors (E‐64d and Pepstatin A) completely stabilized MAVS (Figure 4G; Figure S4G, Supporting Information). Furthermore, these two protease inhibitors restored MAVS to a level that is comparable to transient expression of wild‐type ATP13A1 in *MAVS^−/−^* & *ATP13A1^−/−^* double knockout cells (Figure 4H). Therefore, we concluded that ATP13A1 plays a key role in preventing MAVS from degradation by proteases in the lysosomal pathway.

### 2.5 MAVS Signaling is Impaired in *ATP13A1* ^−/‐^ Cells

**Figure 5:**
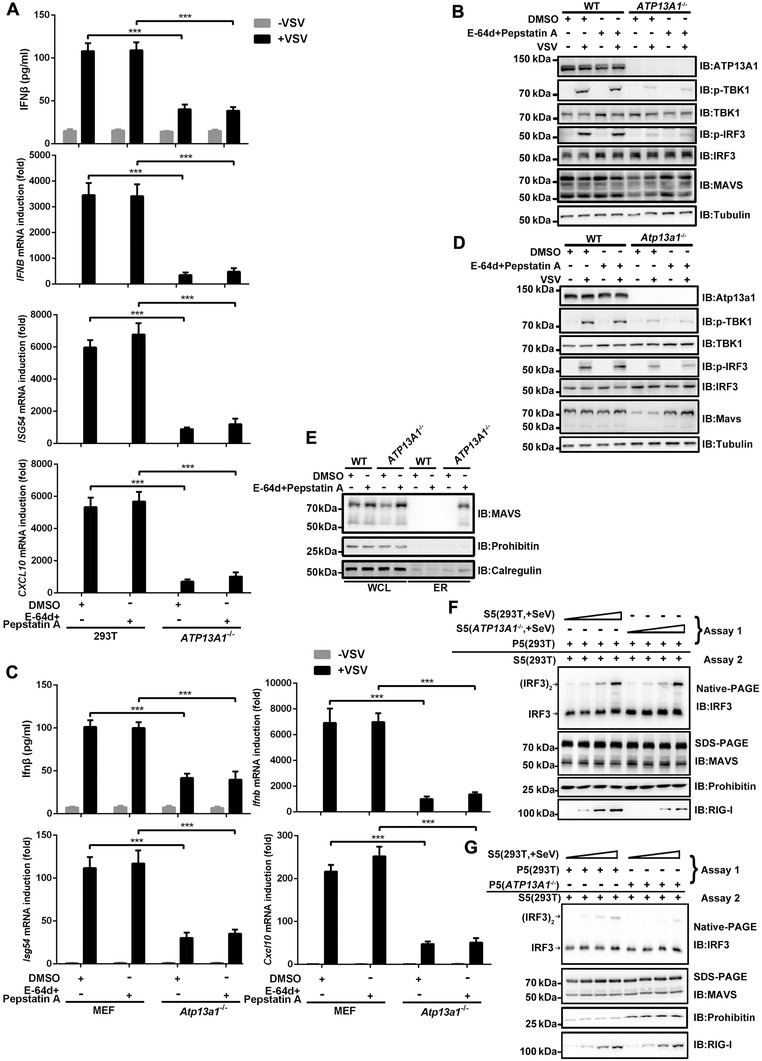
Signaling potential of MAVS is impaired in *ATP13A1* ^−/‐^ cells. A) Wild‐type and *ATP13A1* ^−/−^ HEK293T cells were treated with DMSO or E‐64d (10 µg ml^−1^) and Pepstatin A (10 µg ml^−1^) for 12 h, followed by VSV infection for 12 h before qPCR and ELISA analysis. B) Experiments were performed as described in (A) before immunoblotting analysis. C) Wild‐type and *Atp13a1* ^−/−^ MEF cells were treated with DMSO or E‐64d and Pepstatin A for 12 h, followed by VSV infection for 12 h before qPCR and ELISA analysis. D) Experiments were performed as described in (C) before immunoblotting analysis. E) Wild‐type and *ATP13A1* ^−/−^ HEK293T cells were treated with DMSO or E‐64d and Pepstatin A for 12 h, and ER fractions were isolated respectively before immunoblotting analysis. F) Wild‐type and *ATP13A1* ^−/−^ HEK293T cells were infected with SeV for 8 h and S5 fractions were prepared to incubate with P5 fractions from wild‐type HEK293T cells at 30 °C for 1 h. P5 fractions were then collected after centrifugation, and further incubated with S5 fractions from wild‐type HEK293T cells at 30 °C for another hour. IRF3 dimerization was analyzed by native PAGE. G) HEK293T cells were infected with SeV for 8 h and S5 fractions were prepared to incubate with P5 fractions from wild‐type and *ATP13A1* ^−/−^ HEK293T cells at 30 °C for 1 h. P5 fractions were then separated, and incubated with S5 fractions from wild‐type HEK293T cells at 30 °C for another hour. IRF3 dimerization was analyzed by native PAGE. Data are representative of three independent experiments (shown as mean and SD in A,C). *p* value was determined by two‐tailed unpaired Student's *t*‐test, ***p < 0.001.

We showed that treatment of *ATP13A1* ^−/‐^ HEK293T cells with protease inhibitors E‐64d and Pepstatin A stabilizes MAVS protein. Unexpectedly, restoration of MAVS level did not result in proper antiviral immune response to RNA virus infection, as antiviral genes such as *IFNB*, *ISG54*, and *CXCL10*, could not be induced as expected upon VSV infection (**Figure** **5**A; Figure S5A, Supporting Information). We next examined key molecules in the RIG‐I pathway and found that TBK1 and IRF3 phosphorylation was severely compromised in response to viral infection despite restoration of MAVS level following protease inhibitors treatment (Figure 5B). Similar data were obtained in *Atp13a1‐*deficienct MEF cells (Figure 5C,D). These results were further confirmed in murine primary peritoneal exudate macrophages (PEMs) and bone marrow‐derived macrophages (BMDMs) (Figure S5B‐E, Supporting Information). We then investigated the mechanism that prevents restored MAVS from activating RIG‐I‐MAVS signaling. Previous study in yeast showed that tail‐anchored proteins localized on mitochondrial outer membrane might be accumulated in ER membranes as a result of *SPF1* deletion.^[^ ^15d^ ^]^ Therefore, we speculated the malfunction of restored MAVS could be due to its mislocalization. In wild‐type MEF cells, immunofluorescent staining showed that MAVS is localized in mitochondria but not ER (Figure S5F, Supporting Information). In contrast, after treatment of *ATP13A1‐*deficient cells with protease inhibitors, MAVS is restored and enriched in ER fraction (Figure 5E; Figure S5G, Supporting Information). These data showed that restored MAVS in *ATP13A1‐*deficient cells is mislocalized and could not execute its antiviral function.

Moreover, we noticed that *ATP13A1‐*deficient cells contained some MAVS expression but could not induce antiviral genes upon stimulation. We next compared the activities of MAVS from wild‐type or *ATP13A1‐*deficient cells in an in vitro assay using IRF3 dimerization as a readout. Previous study showed that IRF3 dimerization could be reconstituted in vitro using S5 and P5 fractions isolated from cells.^[^ ^4^, ^19^ ^]^ We first isolated S5 fraction from *ATP13A1* ^−/‐^ HEK293T cells infected with SeV and found that it can activate P5 from wild‐type cells and trigger IRF3 dimerization (Figure 5F), suggesting that the cytosolic machinery of RIG‐I pathway is intact and functional in *ATP13A1* ^−/‐^ HEK293T cells. We then isolated P5 fraction from *ATP13A1* ^−/‐^ HEK293T cells and found that its signaling was dampened in response to stimulation by S5 fraction from wild‐type cells infected with SeV (Figure 5G). Taken together, our data indicate that the remaining MAVS in *ATP13A1‐*deficient cells is functionally impaired and cannot be fully activated or transduce RIG‐I‐mediated antiviral signal effectively.

### 2.6 Atp13a1 is Essential for Antiviral Immune Response in Primary Cells

To investigate the role of Atp13a1 in vivo, we generated *Atp13a1* ^−/−^ mice by CRISPR‐Cas9‐mediated gene editing. *Atp13a1*‐deficiency led to embryonic lethality and all *Atp13a1* ^−/−^ embryos showed growth retardation (Figure S6A–C, Supporting Information). The developmental defect of *Atp13a1* ^−/−^ embryo, as well as the ubiquitous expression of Atp13a1 (Figure S6D, Supporting Information) was indicative of the essential role of Atp13a1. Moreover, we isolated primary cells from *Atp13a1* ^−/−^ embryo and found that *Ifnb* induction was severely impaired upon VSV infection in these cells (Figure S6E, Supporting Information).

**Figure 6:**
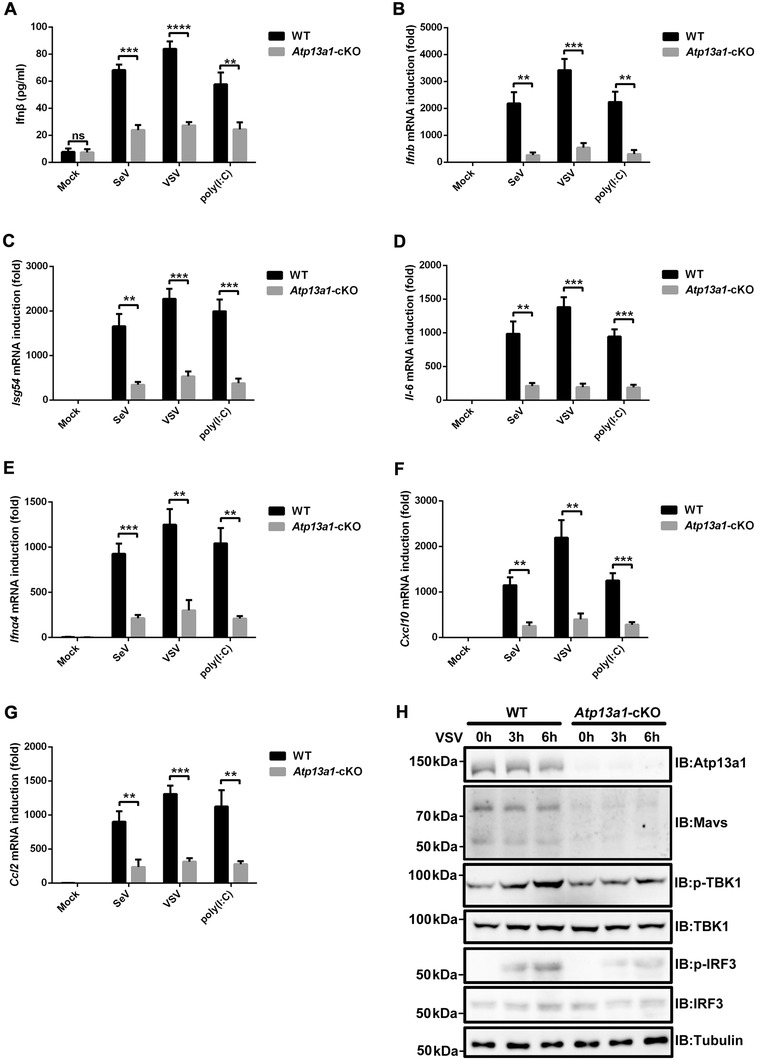
Atp13a1 is essential for antiviral immune response in mouse primary cells. A) Wild‐type and *Atp13a1*‐cKO BMDMs were treated with or without SeV, VSV, or poly (I:C) as indicated for 6 h before ELISA analysis for Ifn*β* in the medium. B–G) Wild‐type and *Atp13a1*‐cKO BMDMs were treated with or without SeV, VSV or poly (I:C) respectively as indicated for 6 h before qPCR analysis for the induction of B) *Ifnb*, C) *Isg54*, D) *Il‐6*, E) *Ifna4*, F) *Cxcl10*, and G) *Ccl2*. H) Wild‐type and *Atp13a1*‐cKO BMDMs were infected with VSV respectively for the indicated time before immunoblotting analysis. Data are representative of three independent experiments (shown as mean and SD in A–G). *p* value was determined by two‐tailed unpaired Student's *t*‐test, **p < 0.01, ***p < 0.001, ****p < 0.0001. ns indicates not statistically significant.

We next generated myeloid‐specific *Atp13a1*‐deficient mice (*Atp13a1*‐cKO) by crossing Lyz2‐cre mice with mice bearing the loxP sites flanking the second and third exons of *Atp13a1*. BMDMs were isolated from *Atp13a1*‐cKO mice and stimulated with poly(I:C) or infected with SeV or VSV. We found that induction of Ifn*β* as well as other antiviral genes including *Isg54*, *Il‐6*, *Ifna4*, *Cxcl10*, and *Ccl2* were dramatically compromised in the absence of *Atp13a1* (**Figure** **6**A–G). We further examined key signaling molecules in the RIG‐I‐MAVS pathway and found that phosphorylation of TBK1 and IRF3 was abolished upon stimulation. Consistent with our data above, MAVS could be barely detected in the absence of Atp13a1 (Figure 6H). Similar results were obtained in PEMs isolated from *Atp13a1*‐cKO mice (Figure S7, Supporting Information). Collectively, these results show that Atp13a1 is required for MAVS stability and a proper antiviral immune response in primary cells including BMDMs and PEMs.

We also investigated whether ATP13A1 is implicated in other immune signaling pathway. LPS, a known agonist for TLR4, stimulates the same amount of Il‐6, IL‐1*β*, and TNF*α* expression in wild‐type PEMs as in *Atp13a1^−/−^* PEMs, suggesting that ATP13A1 may not be involved in TLR4 pathway (Figure S8A,B, Supporting Information). Surprisingly, the cGAS‐STING pathway was compromised in the absence of ATP13A1 when cGAMP was used as a stimulus for STING in *Atp13a1^−/−^* PEMs (Figure S8C, Supporting Information). In contrast to MAVS, protein level of STING was not affected in the absence of ATP13A1 (Figure S8D, Supporting Information). The role of ATP13A1 in cGAS‐STING signaling was validated in BJ cells when HT‐DNA was used as a stimulus (Figure S8E,F, Supporting Information).

### 2.7 Atp13a1 Potentiates Antiviral Immunity in Mice

**Figure 7:**
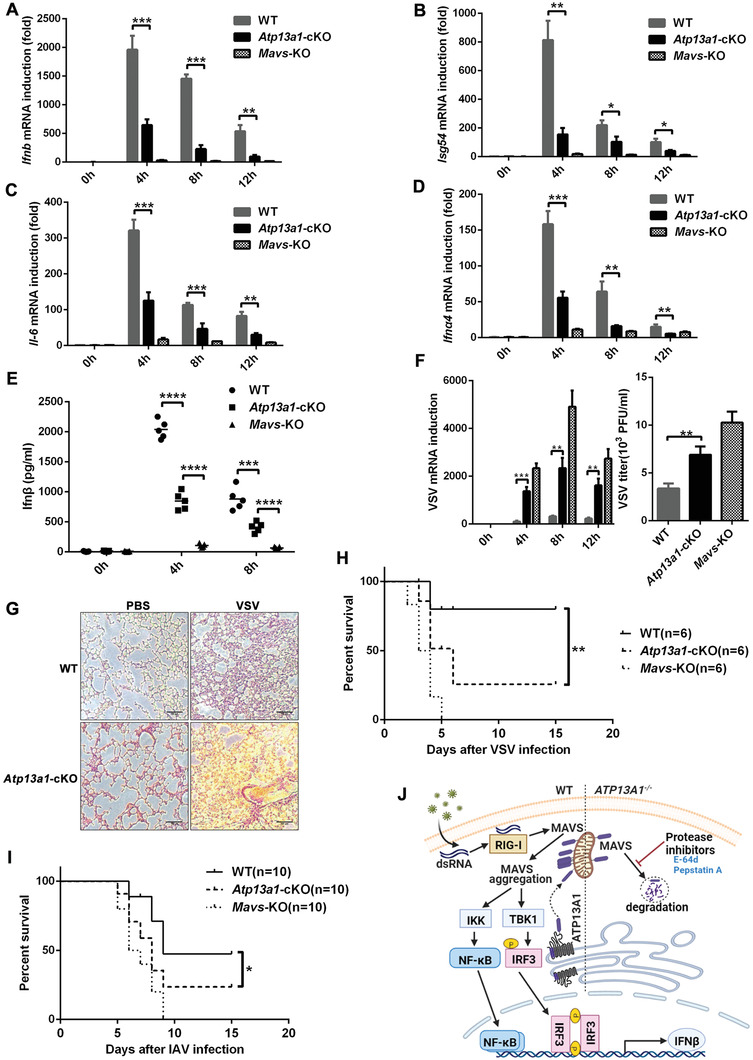
Atp13a1 potentiates antiviral immunity in mice. qPCR analysis of A) *Ifnb*, B) *Isg54*, C) *Il‐6*, D) *Ifna4* induction in spleens from wild‐type, *Atp13a1*‐cKO and *Mavs*‐KO mice (*n* = 3) respectively, following infection for indicated time by intravenous injection of VSV (2 × 10^7^ PFU per mouse). E) Ifn*β* production in serum from mice after intravenous injection with VSV (2 × 10^7^ PFU per mouse) measured by ELISA. F) qPCR analysis for VSV RNA in the spleen from samples as described in (A) (left) and viral titers of spleen following VSV infection for 12 h (right). G) Hematoxylin‐eosin staining of lung sections from the wild‐type and *Atp13a1*‐cKO mice. Scale bar represents 100 micrometers. Survival of the wild‐type, *Atp13a1*‐cKO, and *Mavs*‐KO mice respectively after intravenous injection of H) VSV (5×10^7^ PFU per mouse) or I) IAV (8×10^2^ FFU per mouse). J) A schematic model illustrating functional involvement of ATP13A1 in RIG‐I‐MAVS‐mediated antiviral signaling pathway. The figure was created in BioRender.com. Data are representative of three independent experiments with *n* = 3 (A–D,F), *n* = 6 (H), *n* = 10 (I) technical replicates, each symbol represents an individual technical replicate (E) (shown as mean and SD in (A–D,F); shown as mean and SEM for plaque assay in (F)). *p* value was determined by two‐tailed unpaired Student's *t*‐test (A–F), two‐sided log‐rank (Mantel–Cox) test (H,I), *p < 0.05, **p < 0.01, ***p < 0.001, ****p < 0.0001.

To further investigate the physiological function of Atp13a1 in mice, we challenged wild‐type, *Atp13a1*‐cKO and *Mavs*‐KO mice with VSV. Subsequently, spleens from virus‐treated mice were isolated and induction of *Ifnb*, *Isg54*, *Il‐6*, and *Ifna4* was examined. We found that these antiviral genes were significantly attenuated in *Atp13a1*‐deficienct mice compared to the wild‐type mice (**Figure** **7**A–D). In addition, Ifn*β* in the serum from *Atp13a1*‐cKO mice was much less than that from wild‐type mice (Figure 7E). In line with these results, deficiency of Atp13a1 resulted in increased VSV proliferation in the spleen due to compromised IFN‐I‐mediated antiviral immunity (Figure 7F). Moreover, lungs of *Atp13a1*‐cKO mice showed severe injuries and more infiltration of monocytes than those from the wild‐type mice upon virus infection (Figure 7G). Furthermore, in contrast to the wild‐type control, mice with *Atp13a1* deficiency in macrophages were more susceptible to VSV or IAV infection, resulting in higher mortality following infection (Figure 7H,I). Taken together, our findings demonstrated that Atp13a1 played an important role in IFN‐I‐mediated antiviral innate immunity in vivo.

## 3 Discussion

RIG‐I‐MAVS signaling pathway mediates an important innate immune response against viral infection. In this study, we set up a genome‐wide screen to identify essential molecules in the RIG‐I pathway. We found that ATP13A1, a putative transmembrane helix dislocase on ER that is required to maintain ER and mitochondrial homeostasis, plays a critical role in RIG‐I pathway. ATP13A1 is required both for the maintenance of proper MAVS expression and for the antiviral function of MAVS (Figure 7J).

*ATP13A1*‐deficiency led to severely compromised innate immune response to RNA virus infection in multiple human cell lines and mouse primary cells. Homozygous *Atp13a1* ^−/−^ mice showed growth retardation and embryonic lethality, while myeloid‐specific *Atp13a1* ^−/−^ mice demonstrated no growth defect and were more vulnerable to viral infection than wild‐type mice. Based on these results, we conclude that ATP13A1 is crucial for antiviral innate immunity. Mechanistic analysis indicated that ATP13A1 functions downstream of RIG‐I and upstream of MAVS. Previous reports showed that with viral RNA and ubiquitin chains, antiviral signaling from RIG‐I to MAVS can be reconstituted in vitro, suggesting a unique role of ATP13A1. We found that in the absence of ATP13A1, MAVS was highly unstable, which provided an explanation for the dramatic impairment in antiviral immune response. The reduction of MAVS protein was not at the transcriptional level since its mRNA was not affected by ATP13A1 deficiency. Additionally, the morphology and integrity of mitochondria were not compromised in the absence of ATP13A1 and further analysis revealed that MAVS was the only mitochondrial protein examined with a reduced expression. We deduced that ATP13A1 was specifically required for MAVS stability but not for other mitochondrial proteins.

We further explored the degradation mechanism of MAVS in the absence of ATP13A1. ATP13A1 is the only member of P5A type ATPase family in human, an ER‐localized dislocase with substrates and biological function to be determined. Deficiency of Spf1, the yeast homology to ATP13A1, led to impairment of protein folding and processing. However, we did not detect apparent activation of UPR‐related signaling in *ATP13A1*‐deficient cells, indicating that reduced MAVS level may not be due to UPR pathway. Inhibition of proteasome pathway could not restore MAVS expression, while treatment with protease inhibitors E‐64d and Pepstatin A restored MAVS protein level comparable to that of the wild‐type cells, suggesting that MAVS was degraded mostly by proteases. The proteases responsible for MAVS degradation are most likely derived from host cells rather than viruses, as MAVS can be restored in ATP13A1‐deficient cells following protease inhibitors treatment without virus infection. Unexpectedly, restored MAVS following protease inhibitors treatments could not elicit efficient antiviral immune response. We next examined the subcellular localization of restored MAVS, and found that restored MAVS was localized in ER following E‐64d and Pepstatin A treatments. Mislocalization provides an explanation for the dysfunction of restored MAVS in antiviral response, which is consistent with the previous report that mitochondria localization is critical for MAVS to transduce antiviral signal.^[^ ^2d^ ^]^ ATP13A1 could interact with MAVS to facilitate its translocation from ER to mitochondria. Due to its transmembrane domain, MAVS might be inserted into mitochondrial outer membrane by a previously defined TA‐targeting mechanism.^[^ ^21^ ^]^ In the absence of ATP13A1, MAVS is mislocalized in ER and subjected to protease‐mediated degradation.

Furthermore, we examined the antiviral potential of residual MAVS in *ATP13A1* ^−/‐^ cells with an in vitro assay, which recapitulates the signal transduction from RIG‐I to MAVS and IRF3. The results showed that compared to MAVS from wild‐type cells, residual MAVS from *ATP13A1* ^−/‐^ cells was much less active. In other words, MAVS function to transduce antiviral signal from RIG‐I to TBK1‐IRF3 was severely compromised in *ATP13A1* ^−/‐^ cells, probably due to improper microenvironment on mitochondria or unknown modifications. Taken together, we deduce that ATP13A1 might be required not only for proper subcellular localization of MAVS but also for its antiviral function.

Homozygous *Atp13a1* knock out mice showed growth retardation and embryonic lethality, indicating the importance of *Atp13a1* in development. Defects in eye development are noticeable in *Atp13a1* ^−/‐^ mice and further characterization of the phenotypes awaits extensive investigation. Furthermore, myeloid‐specific *Atp13a1* ^−/−^ mice demonstrated severely compromised antiviral innate immunity, which does not exclude the possible role of ATP13A1 in other lineages or tissues in mediating antiviral innate immune response. Given the critical role of ATP13A1 as the transmembrane helix dislocase at ER, it may be also required for other membrane proteins involved in antiviral response. Indeed, our data suggested that ATP13A1 might be also involved in cGAS‐STING signaling pathway but not in TLR4‐mediated signaling. Therefore, more pleiotropic involvement of ATP13A1 in antiviral immunity is expected.

## 4 Conclusion

Our research reveals that ER‐localized ATP13A1 is essential for RIG‐I‐MAVS signaling, specifically for the stability and activation of MAVS. In the absence of ATP13A1, MAVS is degraded by proteases and the antiviral activity of residual MAVS is compromised.

## 5 Experimental Section

### Animal Experiments

Homozygous *Atp13a1* knockout mice were generated by the CRISPR/Cas9‐mediated genome editing (Shanghai Model Organisms). Genotyping was performed using the primers: forward 5'‐TGGGGAGGATTCCCGAGTAG‐3' and reverse 5'‐GACCAGGAACACAGTAGGGC‐3'. The second and third exons of *Atp13a1* were flanked by two loxP sites through gene editing to generate *Atp13a1* ^fl/fl^ mice, which were then crossbred with Lyz2‐Cre mice to generate *Atp13a1* ^fl/fl^Lyz2‐Cre (termed *Atp13a1*‐cKO) mice. Genotyping was performed using the primers: forward 5'‐CAGATCAGGCTTTGCTTCTCTCTAG‐3' and reverse 5' ‐TGCATGCTACTTTCGTGACACAAG‐3'. In experiments on *Atp13a1*‐cKO mice, the littermates *Atp13a1* ^fl/fl^ (termed WT) were used as controls. *Mavs* knockout mice (*Mavs*‐KO) were generated with the CRISPR–Cas9‐mediated gene editing by Shanghai Model Organisms.^[^ ^8^ ^]^ The genotyping was performed by standard PCR. Mice were maintained in a specific pathogen‐free (SPF) condition, and all animal experiments were carried out in agreement with the regulations of the National Institute of Health Guide for the Care and Use of Laboratory Animals, and approved by the Institutional Animal Care and Use Committee (IACUC) at the Shanghai Institute of Biochemistry and Cell Biology. Permission‐numbers: SYXK 2018‐0007.

### Plasmids and Reagents

Complementary DNA of ATP13A1 was subcloned into pcDNA3‐FLAG expression vector and all mutations were constructed with Fast‐mutagenesis Kit (Vazyme, C214) or overlapping PCR strategy. The primers used in this study are shown in Table S1 (Supporting Information). Other expression plasmids were described previously.^[^ ^4^, ^8^, ^12^ ^]^ Reagents used for the study included Thapsigargin (MCE), LY294002 (Selleck), Bortezomib (Selleck), MG132 (Selleck), 3‐MA (Selleck), Bafilomycin A (Selleck), E‐64d (MCE), Pepstatin A (MCE), and JC‐1 MitoMP detection Kit (Dojindo). Human MAVS antibody was raised by immunizing rabbits with recombinant proteins His‐sumo‐hMAVS‐(aa‐301‐460). Information for commercial antibodies and their dilution in the study is as following: anti‐TBK1 (Cell Signaling Technology, 3504, 1:1000), anti‐RIG‐I (Cell Signaling Technology, 3743, 1:1000), anti‐tubulin (Sigma, T5168, 1:5000), anti‐calregulin (Santa Cruz, SC‐6468, 1:1000), anti‐HA (Cell Signaling Technology, 3724S, 1:3000), anti‐flag (Sigma, F3165, 1:5000), anti‐calnexin (Sigma, C4731, 1:1000), anti‐P62 (Sigma, p0067, 1:3000), anti‐P53 (Cell Signaling Technology, 2524, 1:1000), anti‐VDAC (Proteintech, 10866‐1‐AP, 1:1500), anti‐IRF3 (Abcam, 2241‐1, 1:3000), anti‐ATP13A1 (Proteintech, 16244‐1‐AP, 1:2000), anti‐pTBK1 S172 (Cell Signaling Technology, 5483S, 1:1000), anti‐prohibitin (Abcam, Ab75766, 1:10000), anti‐Fis1 (Proteintech, 10956‐1‐AP, 1:1000), anti‐(mouse) Mavs (Cell Signaling Technology, 4983S, 1:1000) and anti‐pIRF3 S396 (Cell Signaling Technology, 4D4G, 1:1000), anti‐TRAF3 (Santa Cruz, SC‐1828, dilution 1:1000), anti‐TRAF5 (Santa Cruz, SC‐74502, dilution 1:1000), anti‐STAT1 (Abcam, ab109320, 1:10000), anti‐cGAS (Cell Signaling Technology, 66546, 1:1000), anti‐cGAS (ABclonal, A8335, 1:1000) for cells from mice, anti‐STING (ABclonal, A3262, 1:1000), anti‐pSTING (ABclonal, AP1199, 1:1000), anti‐IKK*β* (Abcam, ab32135, 1:1000), anti‐pIKK*α*/*β*(Cell Signaling Technology, 2694, 1:1000), anti‐IΚB*α* (Abcam, ab32518, 1:1000), anti‐pIΚB*α* (Cell Signaling Technology, 9246, 1:1000), anti‐Mfn1 (Beyotime, AF7461, 1:1000), anti‐Mfn2 (ABclonal, A12771, 1:1000), anti‐Bcl‐XL (ABclonal, A0209, 1:1000).

### Cells and Viruses

HEK293T cells, Vero cells, MEF cells, HeLa cells, BJ cells, PEMs (isolated from C57BL/6 mice intraperitoneally injected with 3.0 mL 3% Brewer thioglycollate medium) and BMDMs (isolated from C57BL/6 mice and induced by M‐CSF for seven days) were cultured in DMEM supplemented with 10% FBS, penicillin (100 U ml^−1^) and streptomycin (100 µg ml^−1^). Sendai virus (SeV) was provided by Dr. Xiaozhen Liang (Institute Pasteur of shanghai Chinese Academy of Sciences) and IAV was from Dr. Xiao Su (Institute Pasteur of shanghai Chinese Academy of Sciences). Recombinant virus VSV‐∆M51‐GFP was amplified in Vero cells.

### Generation of I‐5 Cell Line in HEK293T

CRISPR‐Cas9 technique was used to establish GSDMD‐N knock‐in cells. The sgRNA was designed according to the DNA sequence found in National Center for Biotechnology Information Search database (NCBI), and subcloned into the px330 expression vector. Meanwhile, the plasmid IFNB‐P2A‐GSDMD‐N carrying homologous arms was constructed. Vectors were transfected into HEK293T cells using lipofectamine 3000. Puromycin (1 µg ml^−1^) selection was performed for 72 h after transfection. The cells were split and seeded in 96‐well dish. Single‐colony was isolated and I‐5 cells bearing IFNB‐P2A‐GSDMD‐N were verified by Sanger sequencing and immunoblotting. Primers used were shown in Table S1 (Supporting Information).

### Genome‐Wide CRISPR‐Cas9 Screen

The human CRISPR knockout pooled library was purchased from Addgene (GeCKO v2, # 1000000049). Amplification of the library and production of the lentivirus were performed by following the protocol provided by Addgene. I‐5 cells stably expressing Cas9 (I‐5(Cas9)) were established by lentiviral transduction of Cas9 coding sequence. GeCKO v2 library containing 123411 sgRNA targeting 19050 genes was transduced into 1.2 × 10^8^ I‐5(Cas9) cells with an MOI of 0.3. The transduced cells were treated with 1.2 µg ml^−1^ of puromycin. After 7 days, half of the cells were frozen as a control. The rest of the cells were infected with SeV and surviving cells were collected 72 h post‐infection. The genomic DNA of these cells was isolated and the targeted sequences were amplified using Vazyme 2 × Phanta Max Master Mix (P515‐02) by two‐step PCR as described before.^[^ ^20^ ^]^ The PCR products were subsequently isolated from agarose gel and sequenced on HiSeq X10 (Institute of computing technology, Chinese Academy of Sciences).

### Co‐Immunoprecipitation

The expression plasmids with HA or FLAG tag were transfected into HEK293T cells using lipofectamin 2000. After 36 h, cells were harvested and washed for three times with 1×PBS buffer, followed by resuspension with 1×lysis buffer (20 mm Tris‐HCl pH 7.5, 150 mm NaCl, 0.5% Triton X‐100, 1 mm PMSF, protease inhibitor cocktail (Roche)). The cells were then centrifuged at 10000 g for 15 min at 4 °C, and anti‐FLAG M2 beads or anti‐HA beads were added to the supernatant. After incubation for 4 h at 4 °C, the supernatant was removed following centrifugation at 2000 g for 3 min at 4 °C. FLAG or HA beads were washed for three times with lysis buffer and heated at 100 °C for 5 min in 1×SDS loading, which were then subjected to SDS‐PAGE and immunoblotting analysis.

### Immunofluorescence

MEF cells with a density of 20% were seeded in a six‐well plate. After 12 h, cells were incubated with 100 nm MitoTracker Red for 15 min at room temperature. The cells were washed with 1×PBS buffer subsequently, and fixed with 4% paraformaldehyde (PFA) for 15 min at room temperature, followed by one‐time wash with 1×PBS buffer. Cells were then permeabilized with 0.5% TritonX‐100 for 15 min and washed for three times with 1×PBS buffer. After blocking with 3% bovine serum albumin (Roche,10735078001) at room temperature for 1 h, the cells were incubated with anti‐Mavs, anti‐Calnexin, or anti‐Flag antibodies respectively overnight at 4 °C. Then the cells were washed three times with 1×PBS buffer with 0.05% Tween 20 (PBST) and incubated with DAPI (1 µg ml^−1^) and respective secondary antibodies at room temperature for 1 h before fluorescent imaging was taken using Leica TCS SP8. Colocalization of MAVS and ATP13A1 in HeLa cells was observed as described above after transfection with the indicated plasmids for 36 h. Images were background‐subtracted and a region of interest (ROI) was drawn around each cell, after that colocalization was assessed using the ImageJ Coloc2 plugin. Manders’ colocalization coefficients (MCCs) indicate the contribution of HA signal that overlaps with Flag signal normalized to the total HA signal within the ROI. Colocalization of MAVS and ER in MEF cells was analyzed after DMSO or proteases inhibitors treatment as described above.

### RNA Interfering

HEK293T cells or BJ cells were seeded at a confluence of 40%, and 6 h later, si‐*ATP13A1* (GenePharma) was transfected into cells with Lipofectamine RNA iMax (Invitrogen). Forty‐eight hours post‐transfection, cells were harvested and used for following experiments. shRNA was cloned into PLKO.1 and cotransfected with viral packaging vectors into HEK293T cells using Lipofectamine 2000 (Invitrogen). Culture medium containing packaged lentiviral particles was collected after 48 h and incubated with HEK293T cells for 24 h. After puromycin selection, cells were harvested and following experiments were carried out. All sequences of shRNA can be found in Table S1 (Supporting Information).

### Quantitative PCR

Total RNA was extracted using Trizol (TIANGEN) from HEK293T cells, MEF cells, PEMs, or BMDMs. Reverse transcription was performed using HiScript III RT SuperMix for qPCR (+gDNA wiper) (Vazyme, R323‐01). Then qPCR was carried out on Roche Applied Science LightCycler 480 with ChamQ Universal SYBR qPCR Master Mix (Vazyme, Q711‐02/03). The induction fold was determined with the ΔΔCq method and the required qPCR primers used to amplify specific genes were listed in Table S1 (Supporting Information).

### Native‐PAGE and SDD‐AGE

Native‐PAGE was used to analyze IRF3 dimerization as described previously.^[^ ^19^ ^]^ In brief, the samples were mixed with 5×Native loading buffer (100 mm Tris‐HCl pH8.8, 50% Glycerol, 0.04% Bromophenol Blue), and loaded onto a 9% polyacrylamide gel without SDS. After electrophoresis in the running buffer (25 mm Tris‐HCl pH 8.8, 19.2 mm Glycine, 0.4% Doc‐Na) for 60 min with a constant voltage of 200 V at 4 °C, the subsequent procedure was routine western blotting. SDD‐AGE was used to analyze MAVS aggregation. A vertical 1.5% agarose gel was prepared and crude mitochondrial fractions (P5) were obtained by differential centrifugation. P5 was resuspended in 1×SDD loading (10% Glycerol, 0.5×TBE, 2% SDS, and 0.0025% Bromophenol Blue) and loaded onto the agarose gel. Electrophoresis in the running buffer (0.5×TBE, 0.1% SDS) was executed for 40 min with a constant voltage of 100 V at 4 °C, followed by immunoblotting analysis. The signals were visualized by ECL (Share‐Bio, SB‐WB012) and scanned by MiniChemi (SINSAGE).

### IRF3 Dimerization Assay In Vitro

As described previously,^[^ ^12a^ ^]^ HEK293T cells were resuspended by 1×hypotonic buffer (10 mm Tris‐HCl pH 7.5, 1.5 mm MgCl_2_, 10 mm KCL, 0.5 mm EGTA). The cells were homogenized by moving the pestle up and down for 30 times at 4000 rpm. Supernatant (S1) and pellet (P1) were separated by centrifugation at 1000 g for 10 min at 4 °C. The S1 was further fractionated by centrifugation at 10000 g for 10 min at 4 °C, and the supernatant was S5 while the pellet was P5. The indicated fractions were incubated at 30 °C for 1 h and IRF3 dimer was analyzed by Native‐PAGE.

### Endoplasmic Reticulum and MAMs Purification

Endoplasmic reticulum isolation was performed as described previously.^[^ ^22^ ^]^ HEK293T cells were washed with PBS and resuspended with 1×isotonic extraction buffer (10 mm HEPES pH7.8, 25 mm KCL, 250 mm sucrose, 1 mm EGTA, protease inhibitor cocktail (Roche)). The cells were homogenized by moving the pestle up and down for 60 times at 6000 rpm. Supernatant was separated by centrifuging at 20000 g for 30 min at 4 °C, and then mixed with 8 mm calcium chloride solution (CaCl_2_). The ratio of the volume of CaCl_2_ to the supernatant is 7.5. After stirring for 15 min, endoplasmic reticulum fraction was spun down by centrifuging at 8000 g for 10 min at 4 °C. After cellular homogenization, the mitochondria and MAMs are separated by sequential centrifugations as previously described.^[^ ^23^ ^]^

### Plaque Assay

Vero cells were seeded in a 6‐well plate. Culture medium from the infected cells was diluted serially and added to Vero cells. After infection for 1 h at 37 °C, serum‐free medium was removed and replaced with MEM medium containing 0.5% low melting temperature agarose (Lonza) and 10% FBS, followed by incubation for 48 h at 37 °C. The wells were stained with 0.1% crystal violet solution for 5 min at room temperature, and the number of plaque in the plate was counted. Eight‐week‐old mice were infected with VSV (2 × 10^7^ PFU per mouse) by intravenous injection for 12 h, and the spleen were weighed and homogenized three times (5 s each) in PBS. After homogenization, the spleen suspensions were centrifuged at 1,620 g for 30 min, and the supernatants were used for plaque assays.

### ELISA

Concentrations of Ifn*β* (in mouse serum or medium) and IFN*β* (in medium) were measured by ELISA kit for Mouse Interferon *β* (Cusabio, CSB‐E04945m) or Human Interferon *β* (Cusabio, CSB‐E09889h) according to the manufacturer's instructions.

### Statistical Analysis

Two‐tailed unpaired Student's *t*‐test was used to analyze the significance of mean values between groups using Graphpad Prism. Data were presented as mean and standard deviation (SD) or standard error of mean (SEM) as listed in the figure legends. Kaplan–Meier survival analysis was used to analyze mouse survival data by two‐sided log‐rank (Mantel–Cox) test using Graphpad Prism. *p* < 0.05 was considered significant. *p < 0.05, **p < 0.01, ***p < 0.001, ****p < 0.0001, ns indicates not statistically significant.

## Conflict of Interest

The authors declare no conflict of interest.

## Supporting information

Supporting Information

## Data Availability

The data that support the findings of this study are available from the corresponding author upon reasonable request.
