# Supplementary material for: The Endoplasmic Reticulum ATP13A1 is Essential for MAVS‐Mediated Antiviral Innate Immunity
Source: Adv Sci (Weinh). 2022 Oct 10;9(33):2203831. doi: 10.1002/advs.202203831 (PMC9685455; doi:10.1002/advs.202203831)
Supplement: Supplementary file 1 — Supporting Information [file ADVS-9-2203831-s001.pdf]

Supporting information

The Endoplasmic Reticulum ATP13A1 is Essential for MAVS-mediated Antiviral Innate Immunity

*Rui Zhang, Xianteng Hou, Changwan Wang, Jiaxin Li, Junyan Zhu, Yingbo Jiang, and Fajian Hou**

The file includes:

Fig. S1. ATP13A1 regulates RIG-I-MAVS signaling pathway.

Fig. S2. Deficiency of ATP13A1 did not induce ER Stress.

Fig. S3. ATP13A1 is required for maintenance of proper MAVS protein level.

Fig. S4. MAVS is degraded by proteases in the absence of ATP13A1.

Fig. S5. MAVS signaling is impaired in *ATP13A1*$^{-/-}$ cells.

Fig. S6. Atp13a1 regulates antiviral immune response.

Fig. S7. Atp13a1 is essential for antiviral immune response in primary cells.

Figure S8. ATP13A1 is involved in cGAS-STING-mediated antiviral immune response but not TLR4-mediated signaling pathway.

Table S1. Sequence of primers for molecular cloning and Cas9-mediated gene editing or gene knockdown.

Figure S1

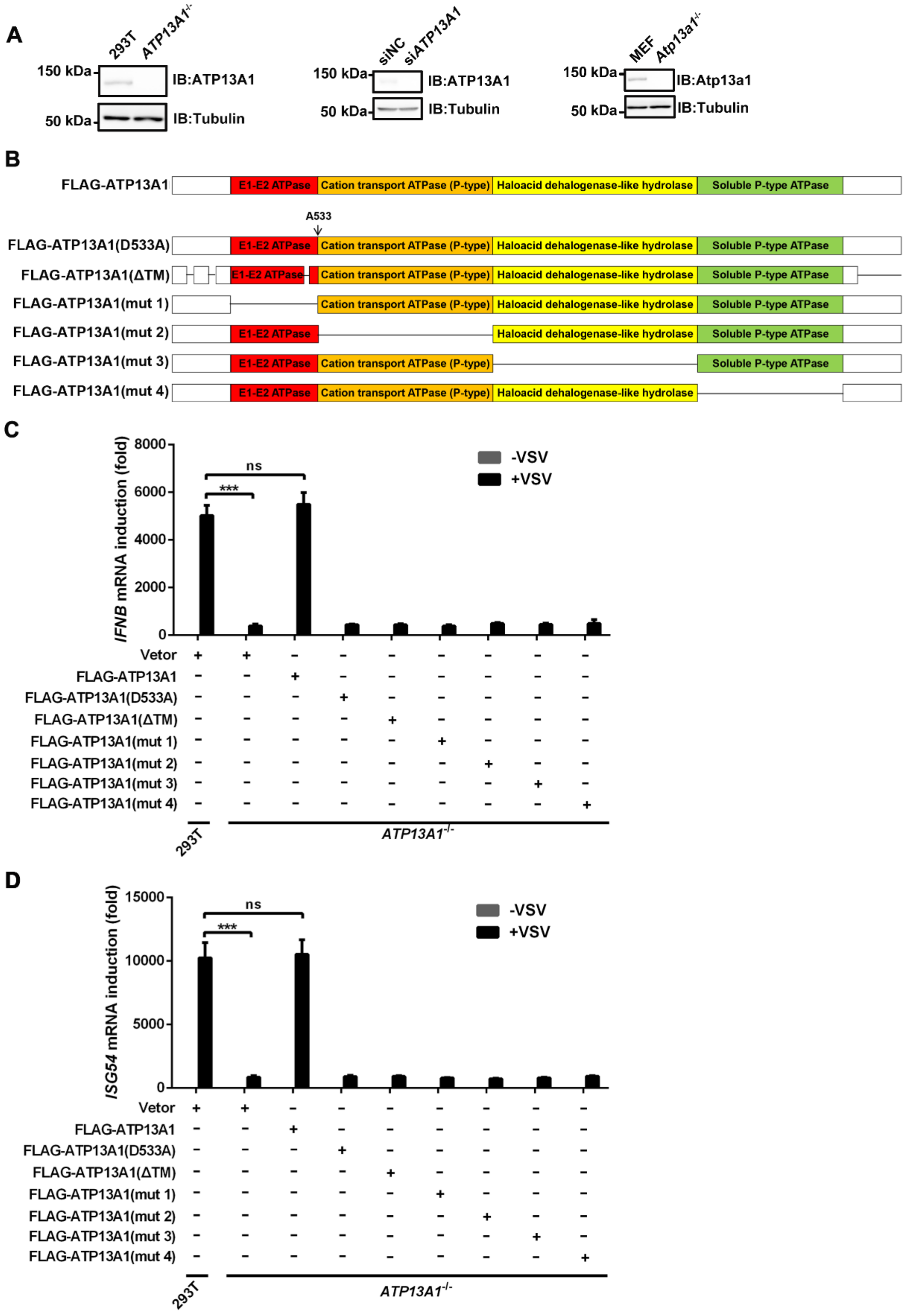
A
293T
ATP13A1-/-
150 kDa
IB:ATP13A1
50 kDa
IB:Tubulin
siNC
siATP13A1
150 kDa
IB:ATP13A1
50 kDa
IB:Tubulin
MEF
Atp13a1-/-
150 kDa
IB:Atp13a1
50 kDa
IB:Tubulin
B
FLAG-ATP13A1
FLAG-ATP13A1(D533A)
FLAG-ATP13A1(ΔTM)
FLAG-ATP13A1(mut 1)
FLAG-ATP13A1(mut 2)
FLAG-ATP13A1(mut 3)
FLAG-ATP13A1(mut 4)
A533
E1-E2 ATPase
Cation transport ATPase (P-type)
Haloacid dehalogenase-like hydrolase
Soluble P-type ATPase
C
IFNB mRNA induction (fold)
8000
6000
4000
2000
0
ns
***
-VSV
+VSV
Vetor
FLAG-ATP13A1
FLAG-ATP13A1(D533A)
FLAG-ATP13A1(ΔTM)
FLAG-ATP13A1(mut 1)
FLAG-ATP13A1(mut 2)
FLAG-ATP13A1(mut 3)
FLAG-ATP13A1(mut 4)
293T
ATP13A1-/-
D
ISG54 mRNA induction (fold)
15000
10000
5000
0
ns
***
-VSV
+VSV
Vetor
FLAG-ATP13A1
FLAG-ATP13A1(D533A)
FLAG-ATP13A1(ΔTM)
FLAG-ATP13A1(mut 1)
FLAG-ATP13A1(mut 2)
FLAG-ATP13A1(mut 3)
FLAG-ATP13A1(mut 4)
293T
ATP13A1-/-

E

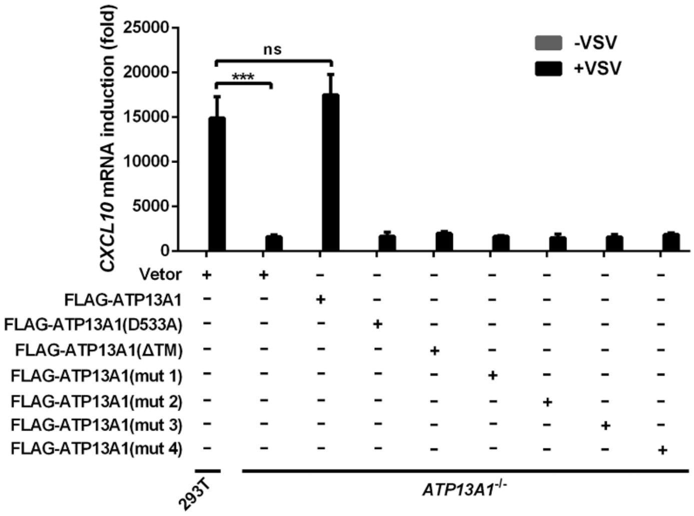

CXCL10 mRNA induction (fold)
25000
20000
15000
10000
5000
0
ns
***
-VSV
+VSV
Vetor
FLAG-ATP13A1
FLAG-ATP13A1(D533A)
FLAG-ATP13A1(ΔTM)
FLAG-ATP13A1(mut 1)
FLAG-ATP13A1(mut 2)
FLAG-ATP13A1(mut 3)
FLAG-ATP13A1(mut 4)
293T
ATP13A1-/-

F

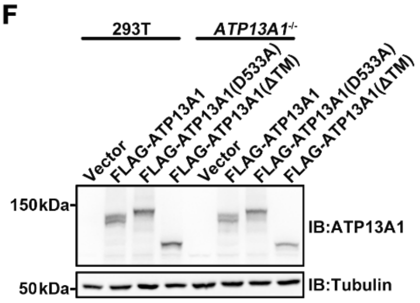

293T
ATP13A1-/-
Vector
FLAG-ATP13A1
FLAG-ATP13A1(D533A)
FLAG-ATP13A1(ΔTM)
Vector
FLAG-ATP13A1
FLAG-ATP13A1(D533A)
FLAG-ATP13A1(ΔTM)
150kDa
50kDa
IB:ATP13A1
IB:Tubulin

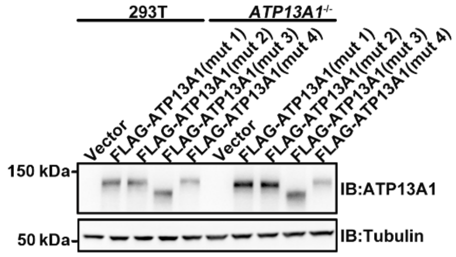

293T
ATP13A1-/-
Vector
FLAG-ATP13A1(mut 1)
FLAG-ATP13A1(mut 2)
FLAG-ATP13A1(mut 3)
FLAG-ATP13A1(mut 4)
Vector
FLAG-ATP13A1(mut 1)
FLAG-ATP13A1(mut 2)
FLAG-ATP13A1(mut 3)
FLAG-ATP13A1(mut 4)
150 kDa
50 kDa
IB:ATP13A1
IB:Tubulin

Figure S1. ATP13A1 regulates RIG-I-MAVS signaling pathway. A) The protein level of ATP13A1 in wild type, *ATP13A1*$^{-/-}$ HEK293T (or *Atp13a1*$^{-/-}$ MEF) cells and *ATP13A1*-knockdown HEK293T cells. B) The diagram of mutation and truncations of ATP13A1. C-E) qPCR analysis for the transcription of *IFNB* (C), *ISG54* (D) and *CXCL10* (E) of *ATP13A1*$^{-/-}$ HEK293T cells transfected with the indicated plasmids. F) Immunoblotting analysis of the wild type and *ATP13A1*$^{-/-}$ HEK293T cells transfected with the indicated plasmids. Data are representative of three independent experiments (shown as mean and SD in C-E). *P* value was determined by two-tailed unpaired Student's *t* test, ***P<0.001. ns indicates not statistically significant.

Figure S2

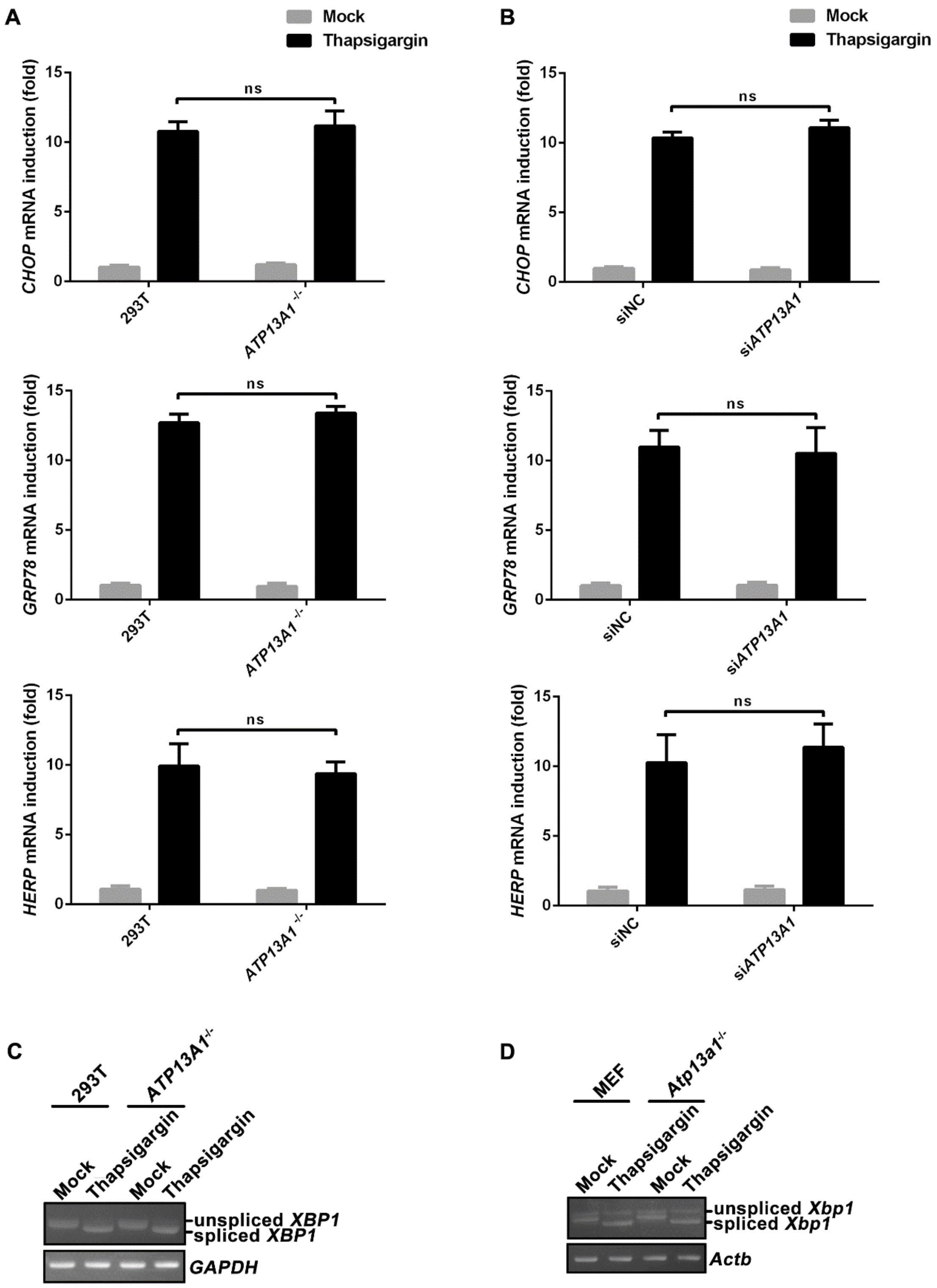
A
B
Mock
Thapsigargin
ns
CHOP mRNA induction (fold)
GRP78 mRNA induction (fold)
HERP mRNA induction (fold)
15
10
5
0
293T
ATP13A1 -/-
siNC
siATP13A1
C
D
293T
ATP13A1-/-
MEF
Atp13a1-/-
Mock
Thapsigargin
unspliced XBP1
spliced XBP1
GAPDH
unspliced Xbp1
spliced Xbp1
Actb

Figure S2. Deficiency of ATP13A1 did not induce ER Stress. A) Wild type and *ATP13A1*$^{-/-}$ HEK293T cells were treated with thapsigargin (0.5 μM) for six hours before qPCR analysis for induction of the indicated genes. B) Wild type and *ATP13A1*-knockdown HEK293T cells were treated with thapsigargin (0.5 μM) for six hours before qPCR analysis for induction of the indicated genes. C) Wild type and *ATP13A1*$^{-/-}$ HEK293T cells were treated with thapsigargin (0.5 μM) for six hours before sliced *XBP1* was separated by agarose gel electrophoresis. D) Wild type and *Atp13a1*$^{-/-}$ MEF cells were treated with thapsigargin (0.5 μM) for six hours before sliced *XBP1* was separated by agarose gel electrophoresis. Data are representative of three independent experiments (shown as mean and SD in A,B). *P* value was determined by two-tailed unpaired Student's *t* test. ns indicates not statistically significant.

Figure S3

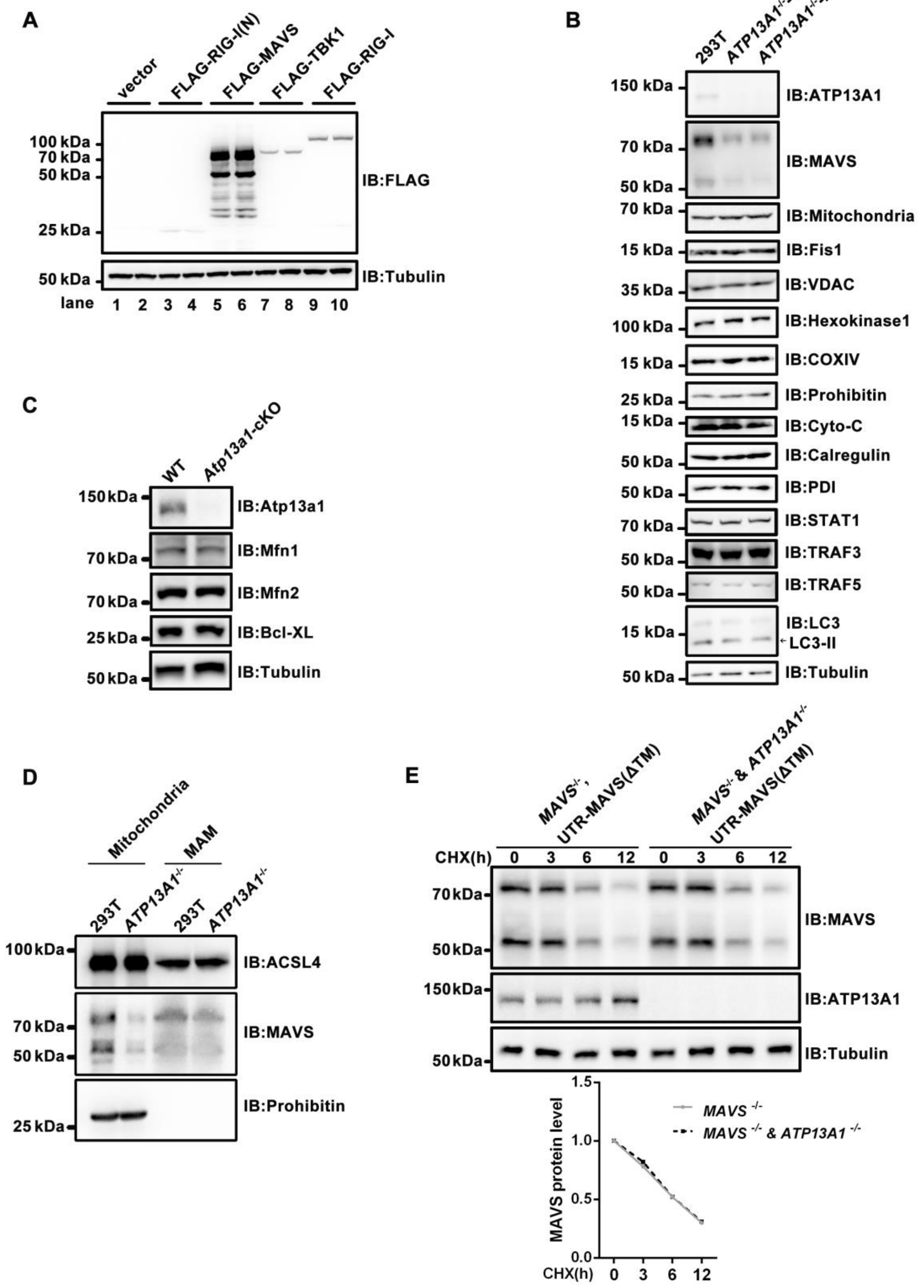
A
vector
FLAG-RIG-I(N)
FLAG-MAVS
FLAG-TBK1
FLAG-RIG-I
100 kDa
70 kDa
50 kDa
25 kDa
IB:FLAG
50 kDa
IB:Tubulin
lane 1 2 3 4 5 6 7 8 9 10
B
293T
ATP13A1-/- -1#
ATP13A1-/- -2#
150 kDa
IB:ATP13A1
70 kDa
50 kDa
IB:MAVS
70 kDa
IB:Mitochondria
15 kDa
IB:Fis1
35 kDa
IB:VDAC
100 kDa
IB:Hexokinase1
15 kDa
IB:COXIV
25 kDa
IB:Prohibitin
15 kDa
IB:Cyto-C
50 kDa
IB:Calregulin
50 kDa
IB:PDI
70 kDa
IB:STAT1
50 kDa
IB:TRAF3
50 kDa
IB:TRAF5
15 kDa
IB:LC3
LC3-II
50 kDa
IB:Tubulin
C
WT
Atp13a1-cKO
150 kDa
IB:Atp13a1
70 kDa
IB:Mfn1
70 kDa
IB:Mfn2
25 kDa
IB:Bcl-XL
50 kDa
IB:Tubulin
D
Mitochondria
MAM
293T
ATP13A1-/-
293T
ATP13A1-/-
100 kDa
IB:ACSL4
70 kDa
50 kDa
IB:MAVS
25 kDa
IB:Prohibitin
E
MAVS-/-,
UTR-MAVS(ΔTM)
MAVS-/- & ATP13A1-/-
UTR-MAVS(ΔTM)
CHX(h) 0 3 6 12 0 3 6 12
70 kDa
50 kDa
IB:MAVS
150 kDa
IB:ATP13A1
50 kDa
IB:Tubulin
MAVS protein level
1.5
1.0
0.5
0.0
CHX(h) 0 3 6 12
MAVS -/-
MAVS -/- & ATP13A1 -/-

Figure S3. ATP13A1 is required for maintenance of proper MAVS protein level. A) The indicated plasmids were transfected into wild type and *ATP13A1*$^{-/-}$ HEK293T cells for twenty-four hours before immunoblotting analysis. Lane 1, Lane 3, Lane 5, Lane 7 and Lane 9 were wild type cell lysates. Lane 2, Lane 4, Lane 6, Lane 8 and lane 10 were *ATP13A1*$^{-/-}$ cell lysates. B) Whole cell lysates of wild type and two *ATP13A1*$^{-/-}$ HEK293T cells were subjected to immunoblotting with the indicated antibodies. C) Whole cell lysates of wild type and *Atp13a1*-cKO BMDMs were subjected to immunoblotting with the indicated antibodies. D) Mitochondrial fractions and MAM fractions from wild type or *ATP13A1*$^{-/-}$ HEK293T cells were analyzed by immunoblotting. E) *MAVS*$^{-/-}$ and *MAVS*$^{-/-}$ & *ATP13A1*$^{-/-}$ double knockout HEK293T cells were transfected with 5'-UTR-MAVS(ΔTM) for 24 hours, and then treated with CHX for the indicated time before immunoblotting analysis (left). Protein level of MAVS normalized to tubulin was quantified (right).

Figure S4

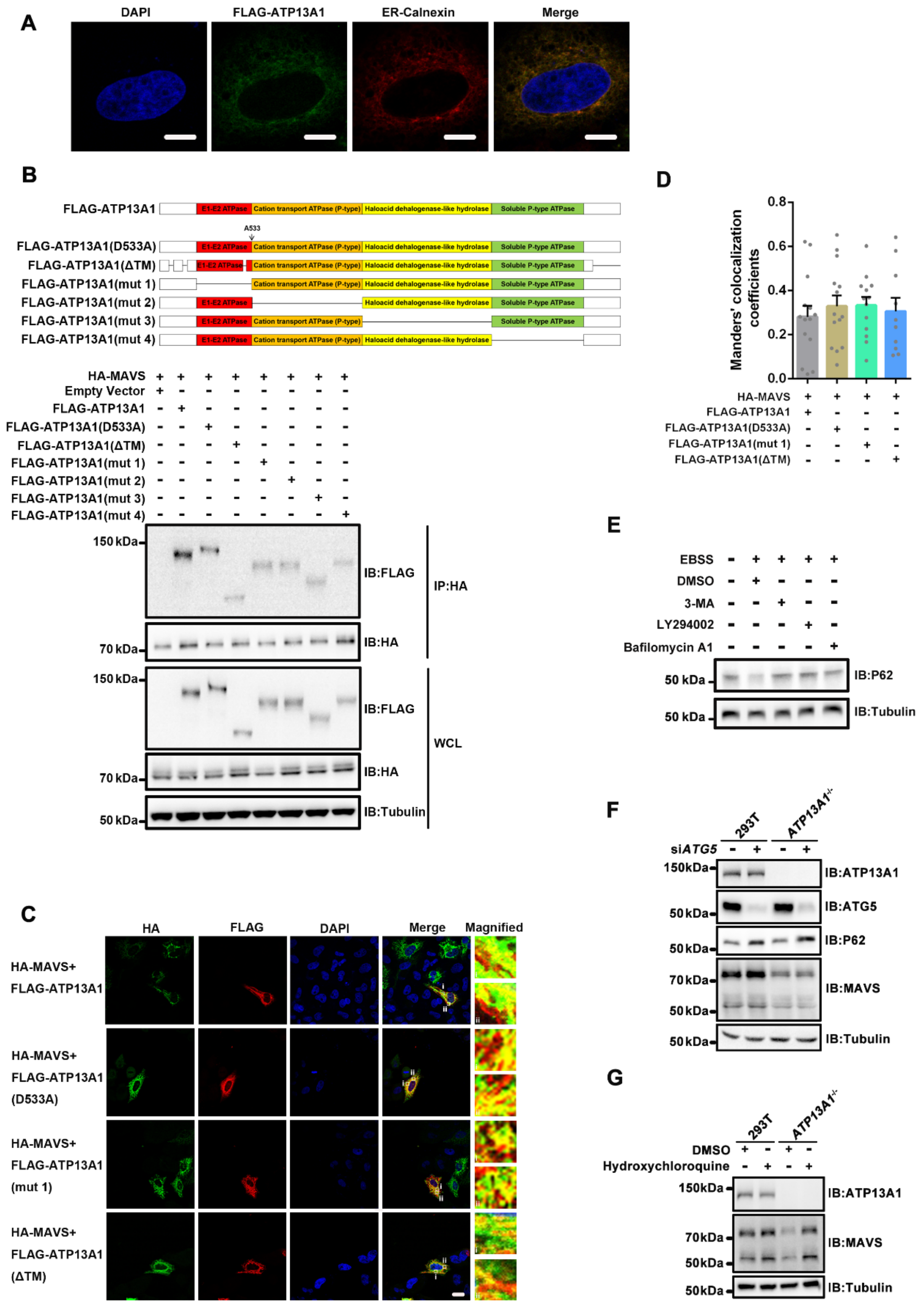
A
DAPI
FLAG-ATP13A1
ER-Calnexin
Merge
B
FLAG-ATP13A1
FLAG-ATP13A1(D533A)
FLAG-ATP13A1(ΔTM)
FLAG-ATP13A1(mut 1)
FLAG-ATP13A1(mut 2)
FLAG-ATP13A1(mut 3)
FLAG-ATP13A1(mut 4)
A533
E1-E2 ATPase
Cation transport ATPase (P-type)
Haloacid dehalogenase-like hydrolase
Soluble P-type ATPase
HA-MAVS
Empty Vector
150 kDa
70 kDa
50 kDa
IB:FLAG
IB:HA
IB:Tubulin
IP:HA
WCL
C
HA
FLAG
DAPI
Merge
Magnified
HA-MAVS+
FLAG-ATP13A1
(D533A)
(mut 1)
(ΔTM)
D
Manders' colocalization coefficients
0.8
0.6
0.4
0.2
0.0
E
EBSS
DMSO
3-MA
LY294002
Bafilomycin A1
IB:P62
F
293T
ATP13A1-/-
siATG5
150kDa
50kDa
70kDa
IB:ATP13A1
IB:ATG5
IB:MAVS
G
Hydroxychloroquine

Figure S4. MAVS is degraded by proteases in the absence of ATP13A1. A) HeLa cells were transfected with FLAG-ATP13A1-expressing vectors. Twenty-four hours after transection, HeLa cells were stained for immunofluorescent microscopic imaging. Nuclei were stained with DAPI. Calnexin (an ER protein) was stained for ER. Anti-FLAG antibody was used for immunofluorescent staining of FLAG-ATP13A1. Scale bar represents 10 micrometers. B) The diagram of various ATP13A1 truncations (upper). HA-MAVS and FLAG-ATP13A1 as indicated were co-transfected into HEK293T cells for thirty-six hours before immunoprecipitation (lower). C) Constructs expressing HA-MAVS and FLAG-ATP13A1 were transfected into HeLa cells. Thirty-six hours after transfection, HeLa cells were stained for immunofluorescent microscopic imaging. Scale bar represents 25 micrometers. D) Individual Manders' colocalization coefficient (MCC) values (per cell) of HA-MAVS colocalization with FLAG-ATP13A1 (WT or mutants). Data were shown as mean and SEM. E) HEK293T cells were cultured in EBSS for four hours, and then cells were treated with the indicated inhibitors before immunoblotting analysis. F) Immunoblotting analysis of cell lysates from wild type or *ATP13A1*$^{-/-}$ HEK293T cells with ATG5 knocked down by siRNA transfection. G) Immunoblotting analysis of cell lysates from wild type or *ATP13A1*$^{-/-}$ HEK293T cells following hydroxychloroquine (10 μM) treatment for twelve hours.

Figure S5

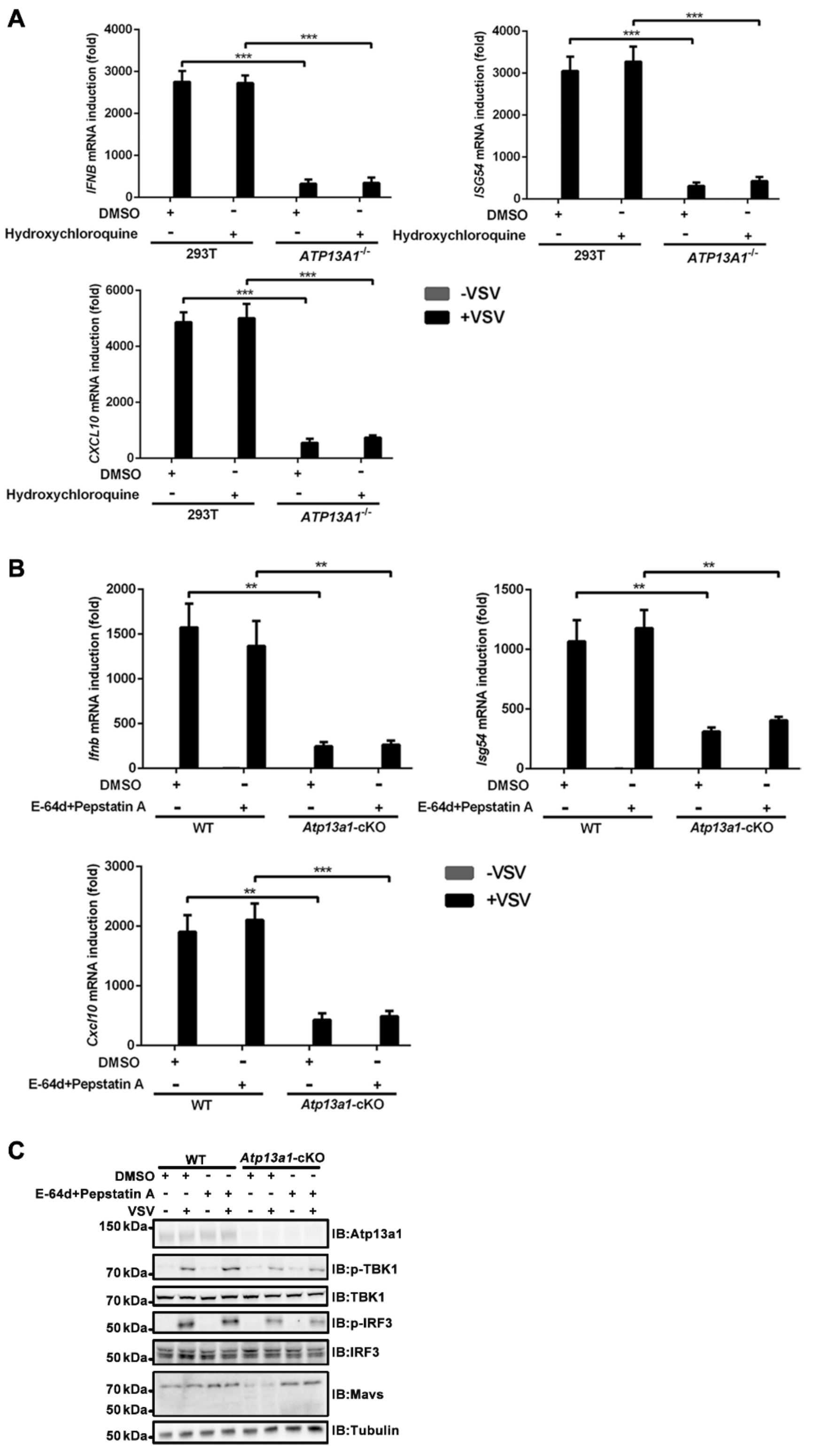

A
IFNB mRNA induction (fold)
ISG54 mRNA induction (fold)
CXCL10 mRNA induction (fold)
DMSO
Hydroxychloroquine
293T
ATP13A1-/-
-VSV
+VSV
B
Ifnb mRNA induction (fold)
Isg54 mRNA induction (fold)
Cxcl10 mRNA induction (fold)
E-64d+Pepstatin A
WT
Atp13a1-cKO
C
VSV
IB:Atp13a1
IB:p-TBK1
IB:TBK1
IB:p-IRF3
IB:IRF3
IB:Mavs
IB:Tubulin

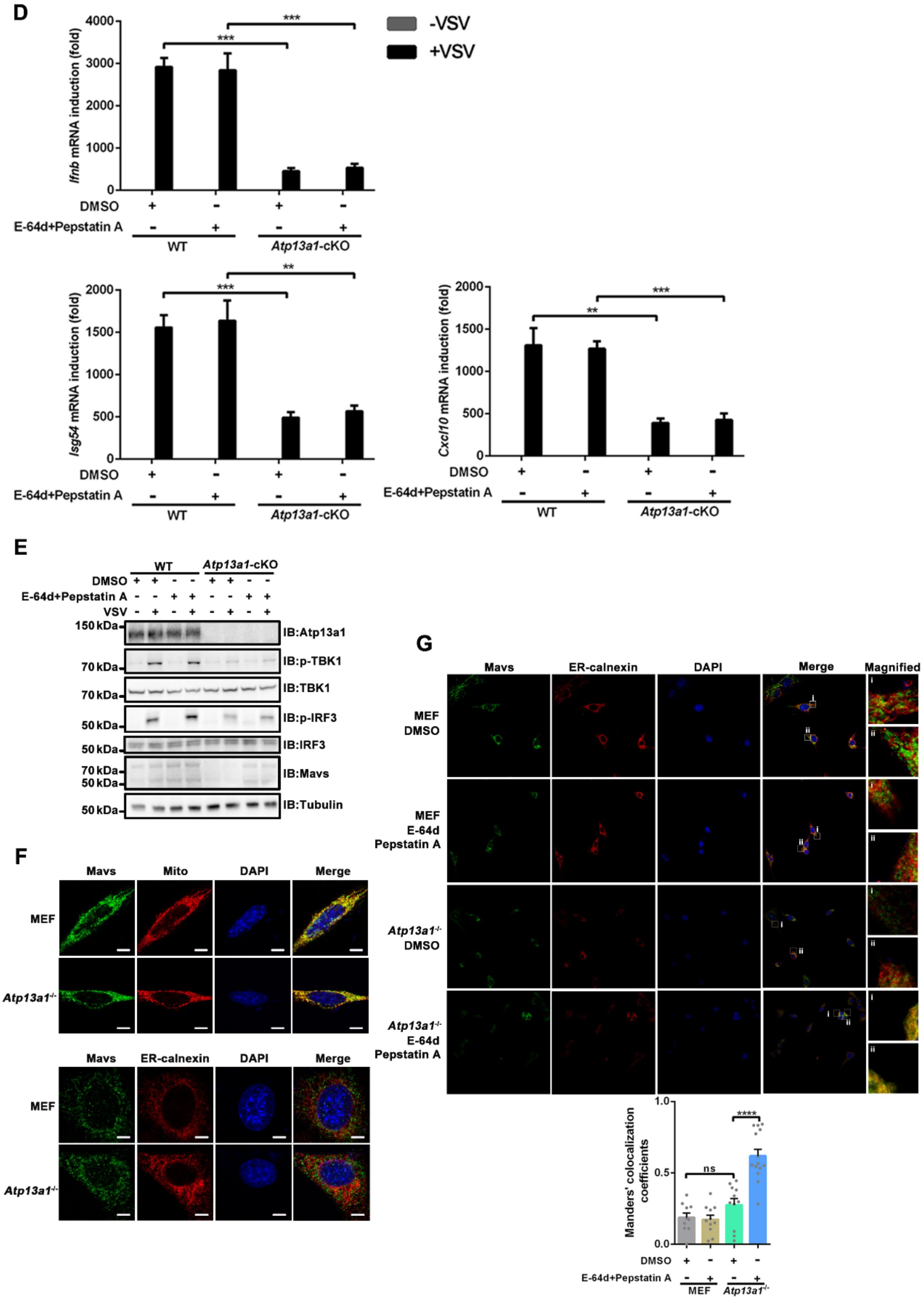

D
-VSV
+VSV
Ifnb mRNA induction (fold)
Isg54 mRNA induction (fold)
Cxcl10 mRNA induction (fold)
DMSO
E-64d+Pepstatin A
WT
Atp13a1-cKO
E
WT
Atp13a1-cKO
DMSO
E-64d+Pepstatin A
VSV
150 kDa
70 kDa
50 kDa
IB:Atp13a1
IB:p-TBK1
IB:TBK1
IB:p-IRF3
IB:IRF3
IB:Mavs
IB:Tubulin
F
Mavs
Mito
DAPI
Merge
MEF
Atp13a1-/-
Mavs
ER-calnexin
DAPI
Merge
MEF
Atp13a1-/-
G
Mavs
ER-calnexin
DAPI
Merge
Magnified
MEF DMSO
MEF E-64d Pepstatin A
Atp13a1-/- DMSO
Atp13a1-/- E-64d Pepstatin A
Manders' colocalization coefficients
ns
****
DMSO
E-64d+Pepstatin A
MEF
Atp13a1-/-

Figure S5. MAVS signaling is impaired in *ATP13A1*$^{-/-}$ cells. A) Wild type and *ATP13A1*$^{-/-}$ HEK293T cells were treated with hydroxychloroquine (10 μM) for twelve hours, followed by VSV infection before qPCR analysis. B,C) Wild type and *Atp13a1*-cKO BMDMs were treated with DMSO or E-64d and Pepstatin A for 12 hours, and then infected with VSV for 6 hours before qPCR analysis for the transcription of the indicated genes (B) and immunoblotting analysis (C). D,E) Wild type and *Atp13a1*-cKO PEMs were treated with DMSO or E-64d and Pepstatin A for twelve hours, and then infected with VSV for six hours before qPCR analysis for the transcription of the indicated genes (D) and immunoblotting analysis (E). F) Wild type and *Atp13a1*$^{-/-}$ MEF cells were stained for immunofluorescent microscopic imaging. Nuclei were stained with DAPI. Mitochondria were stained with MitoTracker Red. Anti-Mavs antibody was used for staining of endogenous Mavs. Calnexin (an ER protein) was stained for ER. Scale bar represents 10 micrometers. G) Wild type and *Atp13a1*$^{-/-}$ MEF cells were treated with or without protease inhibitors for twelve hours and stained for immunofluorescent microscopic imaging. Scale bar represents 25 micrometers. Individual Manders' colocalization coefficient (MCC) values (per cell) of MAVS colocalization with ER were shown as mean and SEM. Data are representative of three independent experiments (shown as mean and SD in A,B,D). *P* value was determined by two-tailed unpaired Student's *t* test, $^{**}P<0.01$, $^{***}P<0.001$, $^{****}P<0.0001$. ns indicates not

statistically significant.

Figure S6

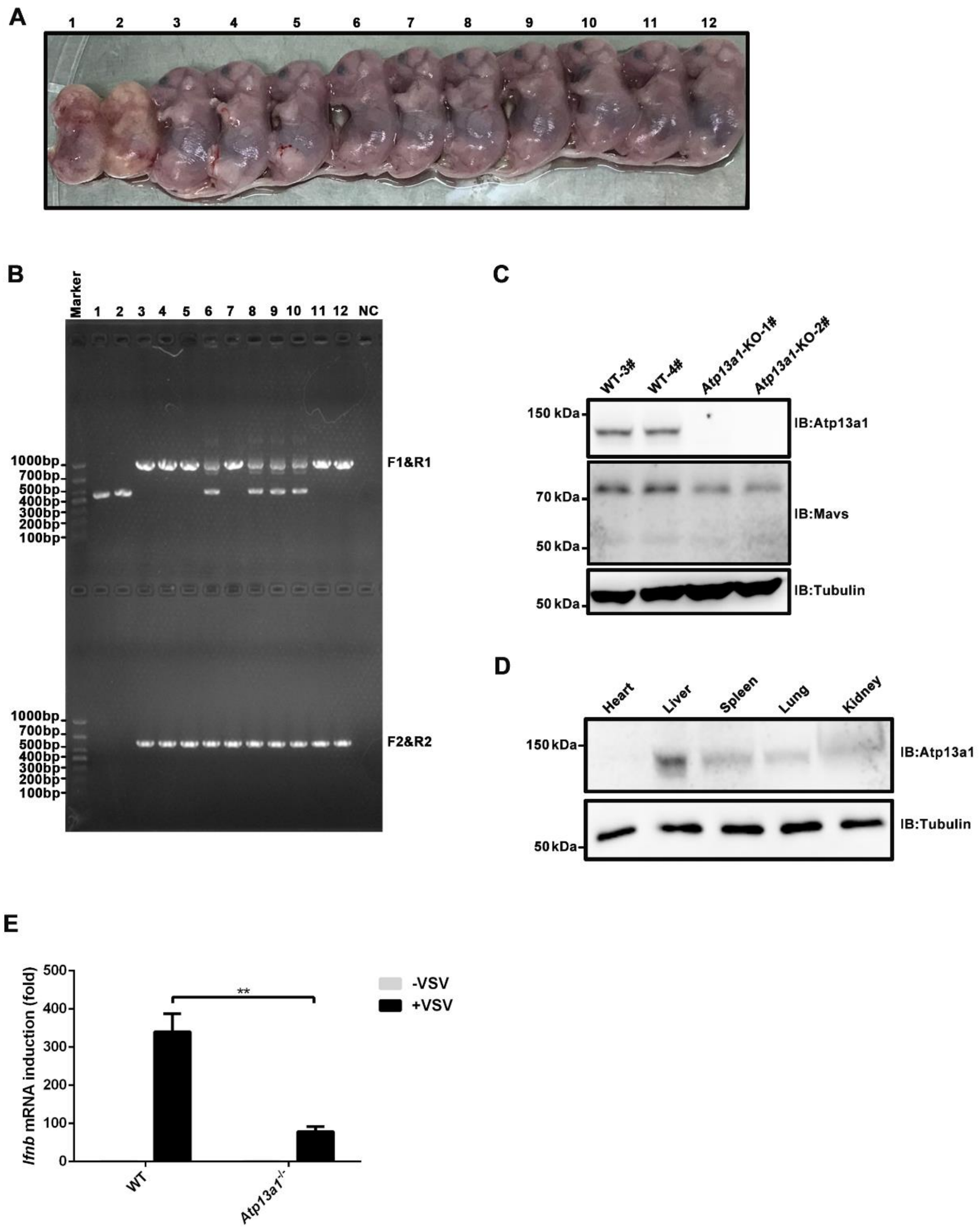

Figure S6. Atp13a1 regulates antiviral immune response. A) The embryos of Atp13a1$^{+/-}$ crossbreed with Atp13a1$^{+/-}$ at Day 18. B) Genotyping of the embryos in (A). C) Immunoblotting analysis of the indicated embryos. D) Immunoblotting analysis of Atp13a1 expression in heart, liver, spleen, lung or kidney from mice. E) Wild type and *Atp13a1*$^{-/-}$ MEF cells from the embryos at day 14 were uninfected or infected with VSV for 8 hours before qPCR analysis for the induction of *Ifnb*. Data are representative of three independent experiments (shown as mean and SD in E). *P* value was determined by two-tailed unpaired Student's *t* test, **P<0.01.

Figure S7

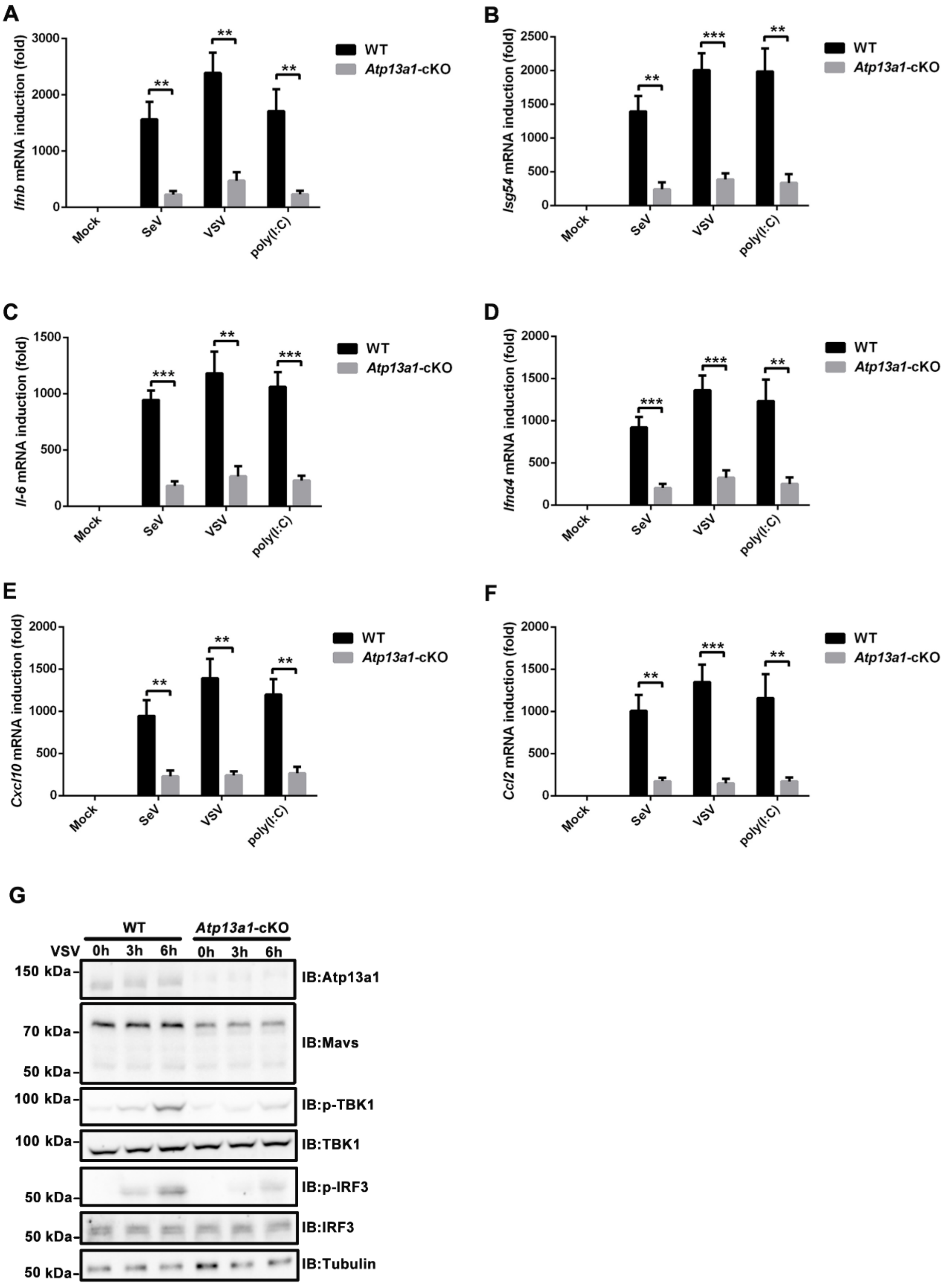

A
B
C
D
E
F
G
Ifnb mRNA induction (fold)
Isg54 mRNA induction (fold)
Il-6 mRNA induction (fold)
Ifnα4 mRNA induction (fold)
Cxcl10 mRNA induction (fold)
Ccl2 mRNA induction (fold)
WT
Atp13a1-cKO
Mock
SeV
VSV
poly(I:C)
VSV
0h
3h
6h
150 kDa
70 kDa
50 kDa
100 kDa
IB:Atp13a1
IB:Mavs
IB:p-TBK1
IB:TBK1
IB:p-IRF3
IB:IRF3
IB:Tubulin

Figure S7. Atp13a1 is essential for antiviral immune response in primary cells. A-F) Wild type and *Atp13a1*-cKO PEMs were treated with or without SeV, VSV or poly (I:C) respectively as indicated for six hours before qPCR analysis for the induction of *Ifnb* (A), *Isg54* (B), *Il-6* (C), *Ifna4* (D), *Cxcl10* (E) and *Ccl2* (F). G) Wild type and *Atp13a1*-cKO PEMs were infected with VSV for the indicated time before immunoblotting analysis. Data are representative of three independent experiments (shown as mean and SD in A-F). *P* value was determined by two-tailed unpaired Student's *t* test, **P<0.01, ***P<0.001.

Figure S8

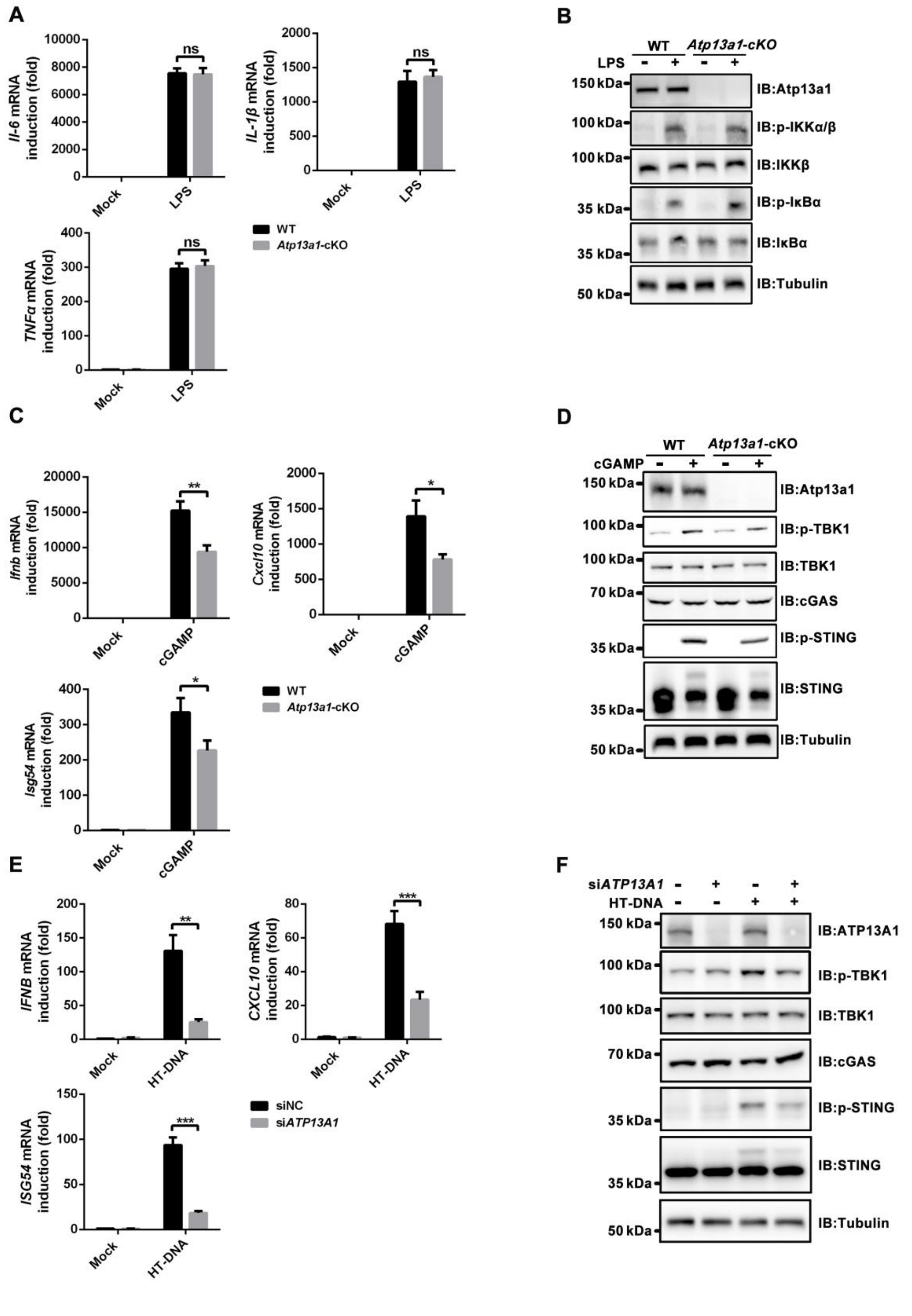
A
Il-6 mRNA induction (fold)
IL-1β mRNA induction (fold)
TNFα mRNA induction (fold)
Mock
LPS
ns
WT
Atp13a1-cKO
B
WT
Atp13a1-cKO
LPS
150 kDa
100 kDa
35 kDa
50 kDa
IB:Atp13a1
IB:p-IKKα/β
IB:IKKβ
IB:p-IκBα
IB:IκBα
IB:Tubulin
C
Ifnb mRNA induction (fold)
Cxcl10 mRNA induction (fold)
Isg54 mRNA induction (fold)
cGAMP
D
70 kDa
IB:p-TBK1
IB:TBK1
IB:cGAS
IB:p-STING
IB:STING
E
IFNB mRNA induction (fold)
CXCL10 mRNA induction (fold)
ISG54 mRNA induction (fold)
HT-DNA
siNC
siATP13A1
F
IB:ATP13A1

Figure S8. ATP13A1 is involved in cGAS-STING-mediated antiviral immune response but not TLR4-mediated signaling pathway. A,B) Wild type and *Atp13a1*-cKO PEMs were stimulated with or without LPS (1 μg/ml) for thirty minutes before qPCR analysis for the induction of *Il-6*, *Il-1β* and *TNF-α* (A) and immunoblotting analysis (B). C,D) Wild type and *Atp13a1*-cKO PEMs were stimulated with or without cGAMP (0.1 μg) for two hours before qPCR analysis for the induction of *Ifnb*, *Isg54*, *Cxcl10* (C) and immunoblotting analysis (D). E,F) The mock or si*ATP13A1*-treated BJ cells were transfected with HT-DNA for twelve hours before qPCR analysis for the induction of *IFNB*, *ISG54* and *CXCL10* (E) and immunoblotting analysis (F). Data are representative of three independent experiments (shown as mean and SD in A,C,E). *P* value was determined by two-tailed unpaired Student's *t* test, $^{*}P<0.05$, $^{**}P<0.01$, $^{***}P<0.001$. ns indicates not statistically significant.

Table S1. Sequence of oligoes for molecular cloning, Cas9-mediated gene editing or mRNA knockdown.

Oligo Name	Sequence(5'-3')	Purpose
ATP13A1-For	AAGCTCGAGATGGCAGCAGCAGCGGCAGTGG	Protein expression
ATP13A1-Rev	GAATAGGGCCCTCTAGATTTCAGGAAGGCACTTTCAGCTTCGG	
ATP13A1-D533A-For	GCTGCTTTGCCAAGACGGGGACGTTGACCAG	
ATP13A1-D533A-Rev	CGTCTTGGCAAAGCAGCACACCTCGACCTTGCC	
ATP13A1-mut 1-For	GCAACAAGCTCTACATGTACTGCACAGAGC	
ATP13A1-mut 1-Rev	GCAGTACATGTAGAGCTTGTTGCCCATCTTCCGGA	
ATP13A1-mut 2-For	ACGGCATCTCCCGGGAAGGAGCCCGCGTC	
ATP13A1-mut 2-Rev	TCCTTCCCGGGAGATGCCGTCGTCCAGCTGCATGA	
ATP13A1-mut 3-For	CGTATGAGAAGCTGCATGCTGACGTGGGTGTGGCG	
ATP13A1-mut 3-Rev	ACCCACGTCAGCATGCAGCTTCTCATACGAGGCAA	
ATP13A1-mut 4-For	TCCGCCTCGCCCCTGAGCGGGTTGTCGA	
ATP13A1-mut 4-Rev	AACCCGCTCAGGGGCGAGGCGGAGCAGCTGCTGG	
ATP-ΔTM-For1	TCCCTCGACGCCATGGACTA	
ATP-ΔTM-Rev1	TGCACAGACAGAGCTAACCGCCGGTACGG	
ATP-ΔTM-For2	TGGCGCTGTCTGTTCATGCGCATTGTGCG	
ATP-ΔTM-Rev2	ATCTGCTGCTTGAAAAGCTCCGAGAAGTCAG	
ATP-ΔTM-For3	TTTCAAGCAGCAGATGCGGAACATGTCG	
ATP-ΔTM-Rev3	AGGGCGATGTTGTTCGCAGTCACCCTTTTGA	
ATP-ΔTM-For4	CGAACAACATCGCCCTCGCTAAGCTGTACAT	

ATP-ΔTM-Rev4	TGAACTATAGAATAGGGCCCTCTAG ATTTC	
IFNB-For	CAGCAGTTCCAGAAGGAGGA	qPCR
IFNB-Rev	AGCCAGGAGGTTCTCAACAA	
GAPDH-For	AGAAGGCTGGGGCTCATTTG	
GAPDH-Rev	AGGGGCCATCCACAGTCTTC	
CXCL10-For	TGGCATTCAAGGAGTACCTC	
CXCL10-Rev	TTGTAGCAATGATCTCAACACG	
ISG54-For	CTGAACCGAGCCCTGCCGAAC	
ISG54-Rev	GCTGCCTCGTTTTGCCCTTTGAG	
CCL5-For	ATCCTCATTGCTACTGCCCTC	
CCL5-Rev	GCCACTGGTGTAGAAATACTCC	
Actin-For (mouse)	TGACGTTGACATCCGTAAAGACC	
Actin-Rev (mouse)	AAGGGTGTAAAACGCAGCTCA	
Ifnb-For (mouse)	CCCTATGGAGATGACGGAGA	
Ifnb-Rev (mouse)	CTGTCTGCTGGTGGAGTTCA	
Isg54-For (mouse)	GGGAAAGCAGAGGAAATCAA	
Isg54-Rev (mouse)	TGAAAGTTGCCATACAGAAG	
Il6-For (mouse)	TCCATCCAGTTGCCTTCTTG	
Il6-Rev (mouse)	GGTCTGTTGGGAGTGGTATC	
Ifna4-For (mouse)	ACCCACAGCCCAGAGAGTGACC	
Ifna4-Rev (mouse)	AGGCCCTCTTGTTCCCGAGGT	
Cxcl10-For (mouse)	GGTCTGAGTGGGACTCAAGG	
Cxcl10-Rev (mouse)	GTGGCAATGATCTCAACACG	
Ccl2-For (mouse)	CCAGCAAGATGATCCCAATG	
Ccl2-Rev (mouse)	TGGTTCCGATCCAGGTTTT	
VSV-For	ACGGCGTACTTCCAGATGG	
VSV-Rev	CTCGGTTCAAGATCCAGGT	
IFNB-P2A-GSDMD-N-For1	ATGAGCAGTCTGCACCTGAAAA	
IFNB-P2A-GSDMD-N-Rev1	AACCATGAGCTTGAGGGCTT	
IFNB-P2A-GSDMD-N-For2	CCAGGGGAAAACTCATGAGCAG	
IFNB-P2A-GSDMD-N-Rev2	CTTCCTAACCACCAGGCAGT	
CHOP-For	GGAAACAGAGTGGTCATTCCC	
CHOP-Rev	CTGCTTGAGCCGTTCATTCTC	
GRP78-For	CATCACGCCGTCCTATGTCG	
GRP78-Rev	CGTCAAAGACCGTGTTCTCG	
HERP-For	CCAGCCCCTATTCACAACCA	

HERP-Rev	CAATAGGGCCACCTTGTGCAT	
ATP13A1-For	GTCTTTACGCTATCCATGCTGG	
ATP13A1-Rev	CACTTGCGGCTTCGGTAGA	
Atp13a1-For (mouse)	CTGGAGGTGCTGTCATTTGAA	
Atp13a1-Rev (mouse)	TCAGCCTTGTTGCTTCCAAAC	qPCR
XBP1-For	TTACGAGAGAAAACTCATGGCC	
XBP1-Rev	GGGTCCAAGTTGTCCAGAATGC	
Xbp1-For (mouse)	GAACCAGGAGTTAAGAACACG	
Xbp1-Rev (mouse)	AGGCAACAGTGTCAGAGTCC	
MAVS-5'-UTR	GGTACCCGAGTCTCGTTTCC	
MAVS-3'-UTR	AGCCAAGGCATGTCCTGCT	
MAVS-For	GACACCCTCTGGCATCTCTTC	
MAVS-Rev	CTTCGTCCGCGAGATCAACTA	
RIG-I-For	GACCCTGGACCCTACCTACA	
RIG-I-Rev	TCCATTGGGCCCTTGTTGTT	
TBK1-For	CGGAGACCCGGCTGGTATAA	
TBK1-Rev	ATCCACTGGACGAAGGAAGC	
Control-sgRNA-For	CACCGACGGAGGCTAAGCGTCGCA A	sgRNA or shRNA
Control-sgRNA-Rev	AAACTTGCGACGCTTAGCCTCCGTC	
IFNB-sgRNA-For	CACCGGCTAGGAGATCTTCAGTTT	
IFNB-sgRNA-Rev	AAACAAACTGAAGATCTCCTAGCC	
MAVS-sgRNA-For	CACCGCTGTGAGCTAGTTGATCTCG	
MAVS-sgRNA-Rev	AAACCGAGATCAACTAGCTCACAGC	
ATP13A1-sgRNA-For	CACCGCGTGGCCCTGCACCGCAAT G	
ATP13A1-sgRNA-Rev	AAACCATTGCGGTGCAGGGCCACGC	
Control-shRNA-For	AATTCTCCGAACGTGTCACGT	
ATP13A1-shRNA-For	CTGACCAAAGATGAGAAAGTA	
SLC10A1-shRNA-For	GCTCACTTATGGAAGCCTAAA	
APLF-shRNA-For	CATCCTGGTGATAGTGATTAT	
CPA4-shRNA-For	GCCGATGTATGTACTGAAGTT	
IRF3-shRNA-For	GCCAACCTGGAAGAGGAATTT	
DDX58-shRNA-For	CCACTTAAACCCAGAGACAAT	
TMEM167A-shRNA-For	GACTGTAATCTTGCTGCTTAT	
SETD8-shRNA-For	GAATCGCAAACTTACGGATTT	
FBP1-shRNA-For	CCTTGATGGATCTTCCAACAT	
MAVS-shRNA-For	CTGCCGCAATTTCAGCAATTT	
